# p14ARF interacts with the focal adhesion kinase and protects cells from anoikis

**DOI:** 10.1038/onc.2017.104

**Published:** 2017-04-24

**Authors:** M Vivo, R Fontana, M Ranieri, G Capasso, T Angrisano, A Pollice, V Calabrò, G La Mantia

**Affiliations:** 1 Dipartimento di Biologia, Università Degli Studi di Napoli ‘Federico II’, Napoli, Italy

## Abstract

The ARF protein functions as an important sensor of hyper-proliferative stimuli restricting cell proliferation through both p53-dependent and -independent pathways. Although to date the majority of studies on ARF have focused on its anti-proliferative role, few studies have addressed whether ARF may also have pro-survival functions. Here we show for the first time that during the process of adhesion and spreading ARF re-localizes to sites of active actin polymerization and to focal adhesion points where it interacts with the phosphorylated focal adhesion kinase. In line with its recruitment to focal adhesions, we observe that hampering ARF function in cancer cells leads to gross defects in cytoskeleton organization resulting in apoptosis through a mechanism dependent on the Death-Associated Protein Kinase. Our data uncover a novel function for p14ARF in protecting cells from anoikis that may reflect its role in anchorage independence, a hallmark of malignant tumor cells.

## Introduction

The ARF protein functions as sensor of hyper-proliferative stimuli restricting cell proliferation through both p53-dependent and -independent pathways.^1^ In line with its tumor-suppressive role, ARF-deficient mice develop lymphomas, sarcomas and adenocarcinomas.^2^ In humans, the importance of ARF inactivation in cancer development is less clear and p16INK4a appears to have a more relevant role in tumor protection.^3^ More than 30 distinct ARF-interacting proteins have been identified, suggesting that ARF is involved in a number of different cellular processes.^4^ Although ARF expression levels in normal proliferating cells are very low, studies based on its loss have revealed its importance in different physiological and developmental mechanisms.^5, 6, 7, 8^

Since its initial discovery, ARF has been described to have a prevalent nucleo-nucleolar localization. More recently, ARF has been reported to localize also in the cytoplasm mainly associated to mitochondria, and also because of its role in autophagy.^9^

Despite its role in growth suppression, ARF is overexpressed in a significant fraction of human tumors.^10^ Increased expression of p14ARF has been associated with progression and unfavorable prognosis in hematological malignancies and in aggressive B-cell lymphomas, and predicts a shortened lifespan.^11^ Furthermore, recent findings suggest that ARF loss hampers, instead of promoting, progression of prostate tumor,^12^ and in mouse lymphomas displaying mutant p53, ARF has been described as having a tumor-promoting activity correlated with its role in autophagy.^13^ Interestingly, it has been reported that the p14ARF protein level increases in thyroid cancer-derived tissues and, remarkably, a delocalization to the cytoplasm has been observed in some aggressive papillary carcinomas.^14^ Although in these cancers ARF has been found to be wild-type, an ARF increase has been explained as accumulation of non-functional protein.

Our previous data suggest that, following activation of protein kinase C, ARF protein is phosphorylated and accumulates in the cytoplasm where it appears unable to efficiently control cell proliferation.^15^ These findings, together with the observations in the cited literature, raise the possibility that ARF expression in cancer cells could aid tumor progression by conferring unknown pro-survival properties to the cells.

Here, we present data showing that during cell adhesion and spreading, p14ARF is delocalized from nucleoli to sites of actin polymerization concentrating at focal contacts where it colocalizes with the focal adhesion kinase (FAK). Moreover, we show that ARF depletion leads to defects in cell spreading and actin cytoskeleton spatial organization in both tumor and immortalized cell lines. Finally, we demonstrate that p14ARF can confer resistance to death-associated protein kinase (DAPK)-dependent apoptosis.

## Results

### ARF localizes to focal contacts during spreading

**Figure 1:**
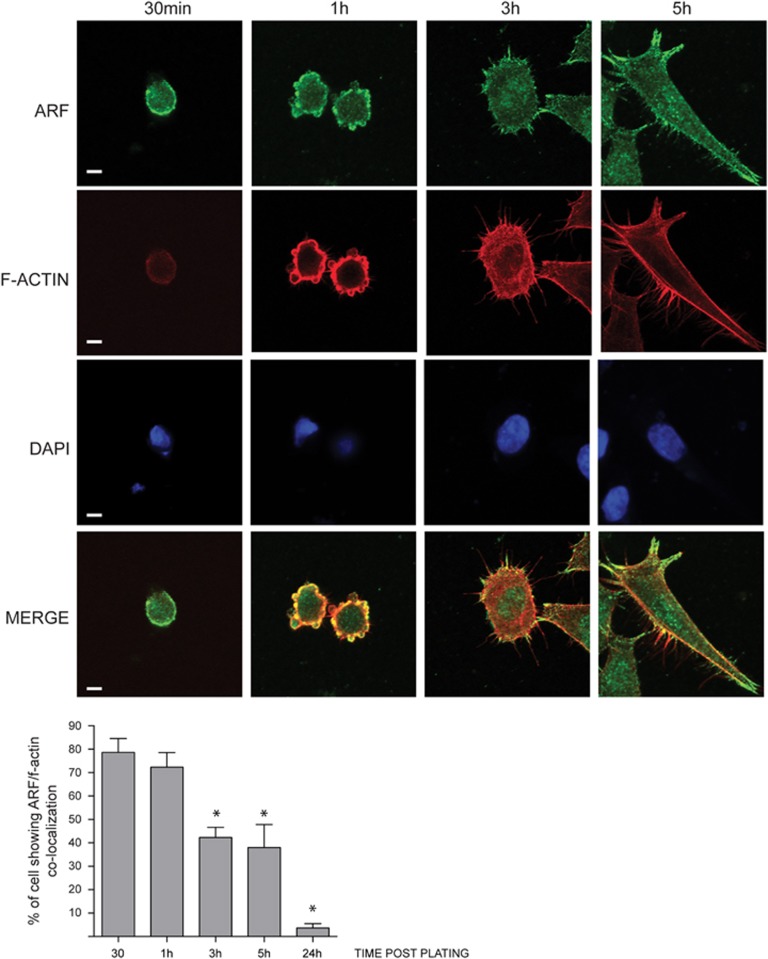
ARF localizes to the plasma membrane during adhesion/spreading process. HeLa cells were detached by gentle trypsin treatment, re-suspended in growth medium and allowed to adhere onto fibronectin-coated coverslip, and at various times following plating, rinsed in PBS (4 °C) and fixed with 3.75% PFA. Cells were fixed at different time points after plating (30’, 1 h, 3 h and 5 h), permeabilized and subjected to IF with anti-ARF antibody and both tetramethylrhodamine phalloidin and DAPI staining to visualize actin cytoskeleton and nuclei. Representative images of ARF subcellular localization are shown for each time point. Images were taken with a Zeiss confocal laser-scanning microscope LSM 510 (Oberkochen, Germany) (scale bar, 7μm). A 40 × objective was used and image analysis was performed using ImageJ. Samples that were to be directly compared were imaged at the same sitting, and the same gain and exposure time were used. Histograms, representing the mean of three independent experiments, reports the percentage of cells in which ARF localize with f-actin. For each time point, 50 cells have been analyzed. S.d. are also shown. Asterisks (*) indicate statistically significant differences by unpaired two-tailed *t*-test with Welch correction: *P<0.001 between each point and 1-h time point.

Cancer-derived HeLa cells express high levels of p14ARF, whereas immortalized HaCaT keratinocytes express low levels of this protein. Remarkably, in HaCaT cells ARF is mainly localized to the cytoplasm.^8^ By immunofluorescence analysis in HeLa and HaCaT cells, we noticed that ARF accumulated at the edge of cells, in particular to lamellipodia and filopodia where rapid actin filament dynamics take place. We therefore examined ARF localization during the process of cellular adhesion and spreading. To synchronize and follow the adhesion process, HeLa cells were detached from the plate by trypsinization, plated onto coverslips and collected at different time points. We analyzed ARF localization by IF (immunofluorescence) while actin cytoskeleton was visualized by tetramethylrhodamine-conjugated phalloidin staining. Thirty minutes after plating, p14ARF was detected along the plasma membrane, (Figure 1; 30 min). During spreading, ARF protein localizes first to cytoplasmic blebs and later on to filopodia (Figures 1, 3 and 5 h after plating). This localization was observed with two different ARF antibodies, and on transfected p14ARF, either tagged with GFP or not (Supplementary Figures S1a–c). Similar results were obtained on cells plated within a three-dimensional substrate such as Matrigel thus suggesting that this localization does not depend on the specific substrate used for adhesion (Supplementary Figure S2a). Immunofluorescence staining of the nucleolar protein B23 showed that nucleoli were not disassembled at any time after seeding (Supplementary Figure S2b).

**Figure 2:**
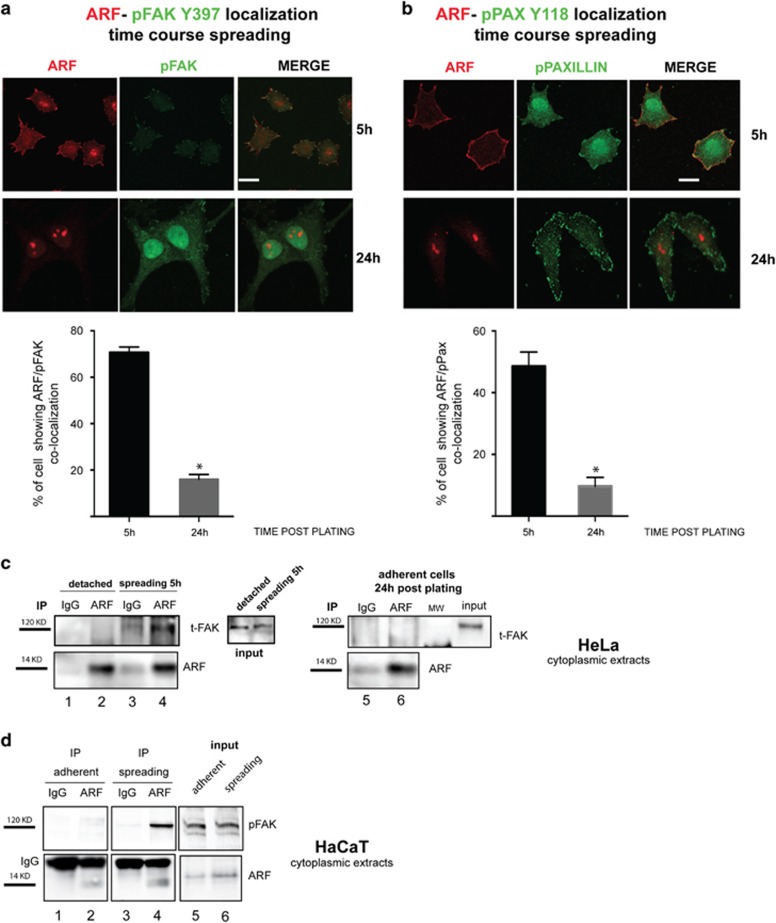
ARF interacts with active FAK during adhesion. ARF colocalizes with active FAK (phosphorylated FAK on Tyr397) and phosphorylated Paxillin during adhesion. HeLa cells were allowed to adhere onto fibronectin-coated coverslips for 5 and 24 h as described before, fixed and subjected to IF with anti-ARF and either pFAK Y397 antibody or pPaxillin Tyr118 antibody. Representative images showing ARF and pFAK (**a**) or ARF and pPaxillin (**b**). Subcellular localization are shown for each time point. Images were taken as described before (scale bar, 10 μm). Histograms below each panel, representing the mean of three independent experiments, reports the percentage of cells in which ARF colocalize with pFAK or pPAXillin Tyr118. S.d. are also shown. Asterisks indicate statistically significant differences (*P*<0.001) as described in Figure 1. ARF and FAK co-immunoprecipitation. (**c**) Cytoplasmic HeLa extracts collected upon cell detachment (lanes 1–2), during spreading (lanes 3–4), and 24 h after adhesion (lanes 5–6) were subjected to immunoprecipitation with anti-ARF antibody or IgG as negative control and western blot with FAK and ARF antibodies. Inputs probed with anti-FAK antibody are shown. (**d**) Same experiment described in **c** performed on HaCaT cells except that IB was performed with anti-pFAK Y397. Molecular weights of both ARF and FAK are shown on the left of each western blot.

Cell interaction with the extracellular matrix results in integrin-mediated tyrosine phosphorylation and recruitment of FAK to focal adhesions.^16, 17^ We thus monitored ARF localization during spreading by following the localization of phosphorylated FAK and of its target, such as Paxillin phosphorylated on Tyrosine 118. Confocal analysis showed ARF/FAK and ARF/Paxillin colocalization at focal adhesions 5 h post-seeding (Figures 2a and b, IF and graph), while 24 h after seeding ARF was mainly localized to the nucleoli. We next performed co-immunoprecipitation experiments of HeLa and HaCaT cytoplasmic extracts during cell spreading (Figures 2c and d) with anti-ARF antibody followed by immunoblot with FAK antibodies. We detected an interaction between p14ARF and FAK 5 h after plating (compare lane 4 with lane 2) that was lost or reduced 24 h after plating (Figure 2c). Incubation with anti-paxillin antibody did not show any interaction with ARF. Reverse co-immunoprecipitation with anti-FAK antibody shows interaction with both ARF and paxillin (Supplementary Figure S3). The experiments thus suggest the existence of a time window during spreading where a transient and specific ARF/FAK interaction takes place at points of focal contacts.

### ARF depletion induces actin cytoskeleton defects and decrease of FAK levels

**Figure 3:**
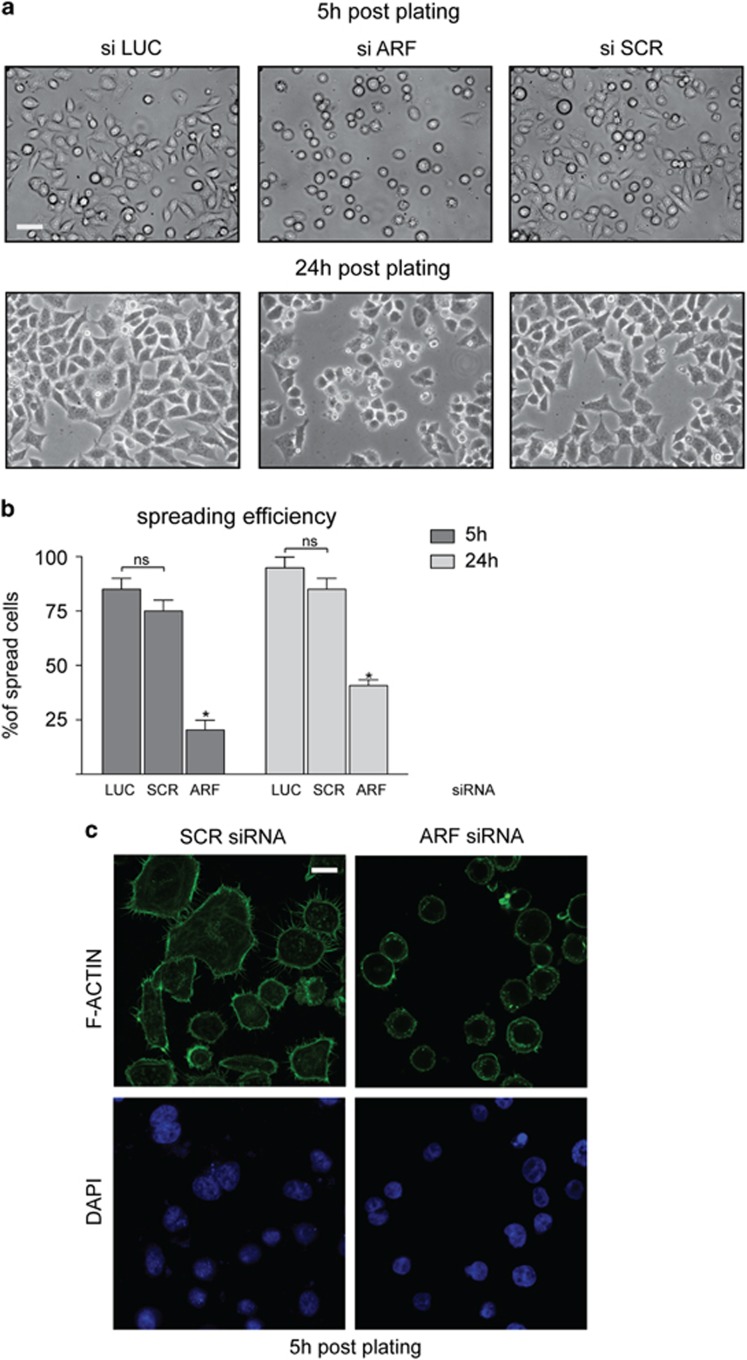
p14ARF depletion affects cell morphology. (**a**) Contrast images of HeLa cells transiently transfected with the indicated stealth siRNAs for 48 h, detached by trypsinization and re-plated at a density of 1 × 10^5^/ml in six-well plates. Images were collected and analyzed by phase-contrast microscope 5 and 24 h after plating with a 20 × objective (scale bar, 30 μm). (**b**) To quantify the percentage of each phenotype, for each transfection point, we counted adherent and round cells in five different fields and pooled data from three to five experiments. Spreading efficiency was measured by assaying the number of adherent cells relative on total cell number (graph). Cumulative data are expressed as a mean value±s.e.m. of three independent experiments. Number of cells analyzed for each experiment: siSCR (200), siARF (200), siLUC (200). Asterisks (*) indicate statistically significant differences by unpaired two-tailed *t*-test between siARF and both siLUC and siSCR: *P<0.0002, whereas NS indicates non statistical significant values. (**c**) Confocal images of HeLa cells treated with control or specific ARF siRNA for 48 h were left to adhere and doubly stained with fluorescein isothiocyanate (FITC)-conjugated phalloidin and DAPI (scale bar, 10 μm).

To analyze ARF role in cell spreading, we transiently transfected cells with three different small interfering RNAs (siRNAs; siARF-1, siARF-2 and siARF-3) targeting different regions of the unique exon 1β of the INK4a/ARF locus (Supplementary Figure S4a). As control, we found that p16INK4a levels were not significantly affected upon ARF silencing (Supplementary Figures S4a and b). Control and ARF-depleted cells were then detached and re-plated to follow the spreading process over time by phase-contrast microscopy. Five hours after seeding, control cells were almost completely spread, whereas ARF KD (Knock-Down) cells exhibited a clear round morphology (Figure 3a and Supplementary Figure S4c). The same effect was seen at 24 h after plating (Figure 3a). Quantification of spreading efficiency showed that less than 50% of ARF-depleted cells were able to properly spread (Figure 3b) As control, silencing of p16INK4a by RNA interference does not affect cell morphology (Supplementary Figure S4d). Staining with fluorescein isothiocyanate-conjugated phalloidin showed that ARF KD cells present blebs around the cell periphery and rare or shorter filopodia (Figure 3c). Similar results were obtained in both HeLa and HaCaT cells grown on Matrigel (Supplementary Figures S5a and b)

**Figure 4:**
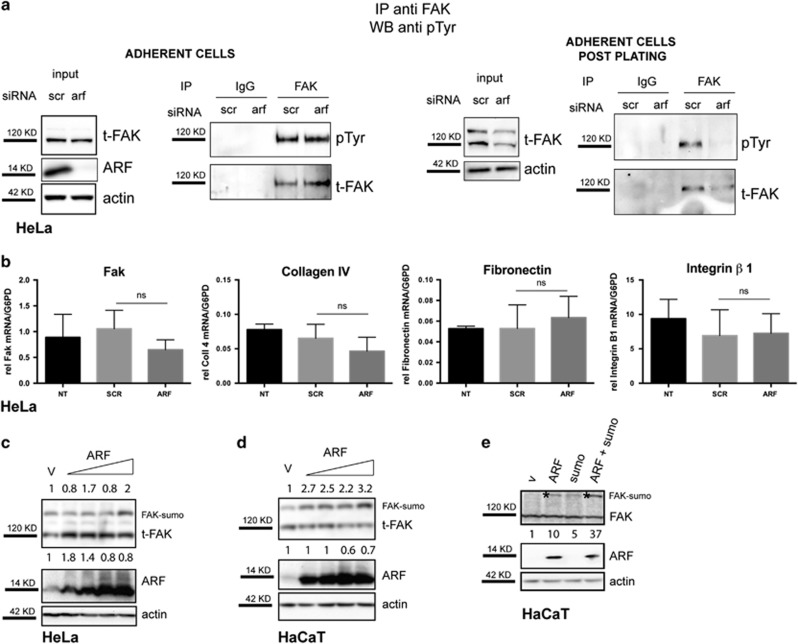
ARF depletion affects FAK activation. (**a**) HeLa total extracts of ARF and control-depleted cells (adherent cells) were immunoprecipitated with anti-total FAK antibody or with IgG as negative control and analyzed by western blot with anti-pTyr antibody and subsequently with anti-t-FAK antibody cells before (left panels) or after adhesion following detachment and re-plating (right panels). Panels of inputs probed with anti-FAK, ARF and actin antibodies are also shown on the left of each immunoprecipitation experiment. (**b**) Quantitative reverse transcriptase (qRT)–PCR analyses of FAK, collagen IV, fibronectin and integrin β1 mRNA levels normalized on G6PD expression in cells treated with the indicated siRNA are shown. Cumulative data are expressed as a mean value±s.d. of three independent experiments. Statistical analysis performed by unpaired, two-tailed Mann–Whitney test show no statistical (NS) difference between siSCR and siARF samples. (**c**) HeLa and HaCaT cells were transfected with an empty vector (v) and increasing amount of p14ARF expression plasmid. Twenty-four hours after transfection, cells were lysed and cellular extracts were immunoblotted and analyzed with anti-FAK, anti-ARF antibody to detect exogenous ARF levels and anti-actin as loading control. (**d** and **e**) HaCaT cells were transfected with plasmids encoding ARF proteins alone or in combination with plasmid encoding SUMO protein. Cellular extracts were analyzed as described before in **b**. Asterisks indicate slow migrating FAK species. Western blots shown are representative of at least three independent experiments. Normalized FAK band intensities are shown below each corresponding band, whereas sumo FAK are indicated on top above, and are expressed as fold enrichment with respect to empty vector-transfected sample arbitrarily set to 1 (see Materials and Methods section for details).

As it was previously shown that defects in FAK phosphorylation induces cells to acquire a round morphology,^18, 19^ we analyzed the levels of phospho-FAK in ARF-depleted cells by FAK immunoprecipitation and phospho-Tyr immunodetection in HeLa cells not subjected to re-plating (Figure 4a, left panels), and upon detachment and re-plating (Figure 4a, right panels). No significant change in FAK tyrosine phosphorylation occurred upon ARF KD before re-plating. Instead, we found a decrease of FAK tyrosine phosphorylation mirrored by total FAK levels reduction when ARF KD cells were induced to spread. Quantitative reverse transcriptase–PCR analysis showed no significant change in FAK mRNA levels upon ARF depletion, neither in the expression levels of genes involved in cell adhesion such as fibronectin, type IV collagen and integrin β1 (Figure 4b). In agreement with this, transfection of increasing amounts of ARF-expressing plasmid induces an increase in the protein levels of FAK in HeLa cells (Figure 4c). In addition to the 120 kDa FAK immunoreactive band, slow migrating species, previously demonstrated to correspond with SUMO-conjugated FAK forms,^20^ appear to be stabilized upon ARF expression (Figure 4c, last lane). This is particularly evident in HaCaT cells, where ARF overexpression induces a strong increase of slow migrating FAK species (Figure 4d), and upon ARF and SUMO co-transfection (Figure 4e) thus suggesting that ARF could mediate FAK stabilization through sumoylation.^21^

### ARF ectopic expression rescues both morphology and FAK phosphorylation defects of ARF-depleted cells

**Figure 5:**
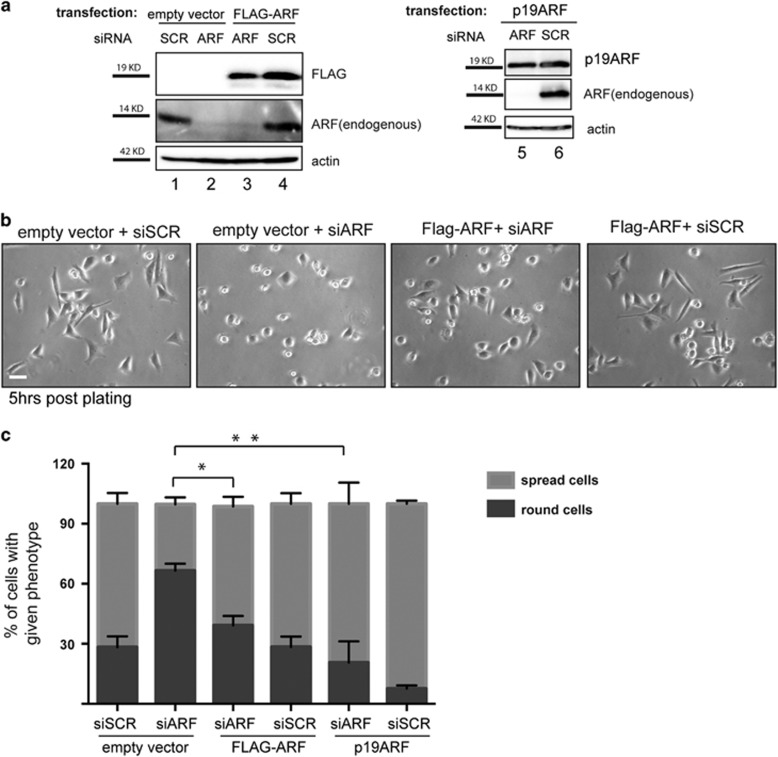
Both human and mouse ARF transfections rescue cell spreading defect of p14ARF-depleted cells. HeLa cells transiently transfected with empty, flag-ARF or p19ARF vector for 24 h were treated with the indicated siRNAs for 48 h, detached and re-plated as described above. (**a**) Western blot analysis with anti-Flag, ARF (human and mouse) and actin from collected cells. (**b**) Images of re-plated cells acquired by phase-contrast microscope (scale bar, 30μm). (**c**) Cumulative data are expressed as a mean value±s.e.m. of at least three independent experiments. Single experiment data have been tested as described in Materials and methods section to analyze normal distribution. Asterisks (*) indicate statistically significant differences by paired two-tailed Wilcoxon test. *P<0.001 and **P<0.01 by paired Student's *t*-test. Number of cells analyzed for each experiment: SCR siRNA+ empty vector (350), ARF siRNA+empty vector (330), ARF siRNA+Flag-ARF (300), SCR siRNA+Flag-ARF (350), SCR siRNA+p19ARF (336) and ARF siRNA+p19ARF (300).

To test if the effects of ARF silencing observed in HeLa cells were strictly dependent on ARF, we performed a rescue experiment transfecting both human and mouse ARF protein. Upon transfection with either an empty or an ARF-expressing vector, cells were treated with scrambled or ARF-specific siRNA-1 for 48 h and then induced to spread. Both p14ARF and p19ARF expression rescued spread morphology defect caused by ARF silencing (Figure 5) thus suggesting the existence of an evolutionary conserved mechanism. In line with this, mouse protein also shows f-actin co-localization during spreading (Supplementary Figure S6a).

**Figure 6:**
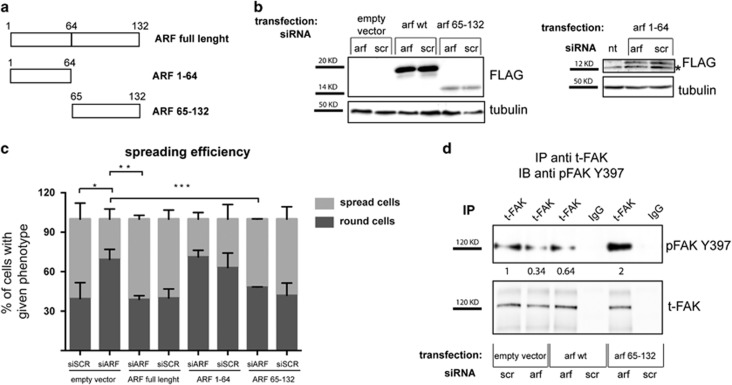
Re-introduction of ARF exon 2 encoded domain in ARF-depleted cells restore both spreading and pFAK expression. Rescue experiment was performed as described before, except that siRNA for the 5’-UTR of ARF gene was used (siRNA-3), and different ARF tagged forms were tested. (**a**) Scheme of N-ter flagged forms of ARF used in this study. (**b**) Immunoblot analysis of flagged 14 ARF mutants and tubulin (loading control) expression levels in whole-cell lysates of cells transfected and silenced with the indicated plasmids and siRNAs. Asterisks indicate an aspecific flag band. (**c**) Quantification of spreading efficiency of cells. Data were quantified from three or more experiments and 400 cells were analyzed for each transfection point in every experiment. Error bars represent the s.d. from the mean and statistical analysis performed as described in Figure 5. *P<0.02 siARF-empty vector vs siSCR-empty vector; **P<0.0092 siARF-Flag-ARF vs siARF-empty vector; ***P<0.0112 siARF-Flag 65–132 vs siARF-empty vector. (**d**) Analysis of pFAK and total FAK intracellular protein levels upon rescue experiment by IP with anti-t-FAK antibody and IB with anti-pFAK Y397 antibody performed as previously described. Representative western blots are shown. pFAK band intensities normalized versus t-FAK are shown below each corresponding band, and are expressed as fold value with respect to empty vector siSCR-transfected sample, arbitrarily set to 1 (see Materials and Methods section for details).

To map ARF region responsible of this function, we performed a rescue experiment by transfecting Hela cells with different ARF deletion mutants corresponding to either aa 1–64, encoded by the exon 1β, widely demonstrated to be necessary to elicit ARF cell cycle arrest,^22, 23, 24^ or aa 65–132, encoded by the exon 2 (Figures 6a and b). Transfected cells were treated with luciferase or ARF-specific siRNA targeting the 5’-UTR region of endogenous transcript (siARF-3). As both ARF mutants migrate with approximately the same molecular weight of the endogenous protein, the silencing of endogenous gene was checked in cells transfected with the empty vector (data not shown). The evaluation of the spreading efficiency (Figure 6c) clearly indicated that the ARF region encoded by exon 2 was able to rescue the spreading defect induced by depletion of the endogenous protein. Regarding the exon 1β encoded domain, we noticed that its expression *per se* (1–64 siSCR) impairs spreading similarly to ARF depletion, and treatment with ARF siRNA (1–64 siARF) does not further affect cell spreading.

We next tested 65–132 mutant and p19ARF ability to rescue FAK phosphorylation by FAK immunoprecipitation followed by phospho-FAK immunodetection and observed that both proteins restore normal levels of active FAK (Figure 6d and Supplementary Figure S6b).

**Figure 7:**
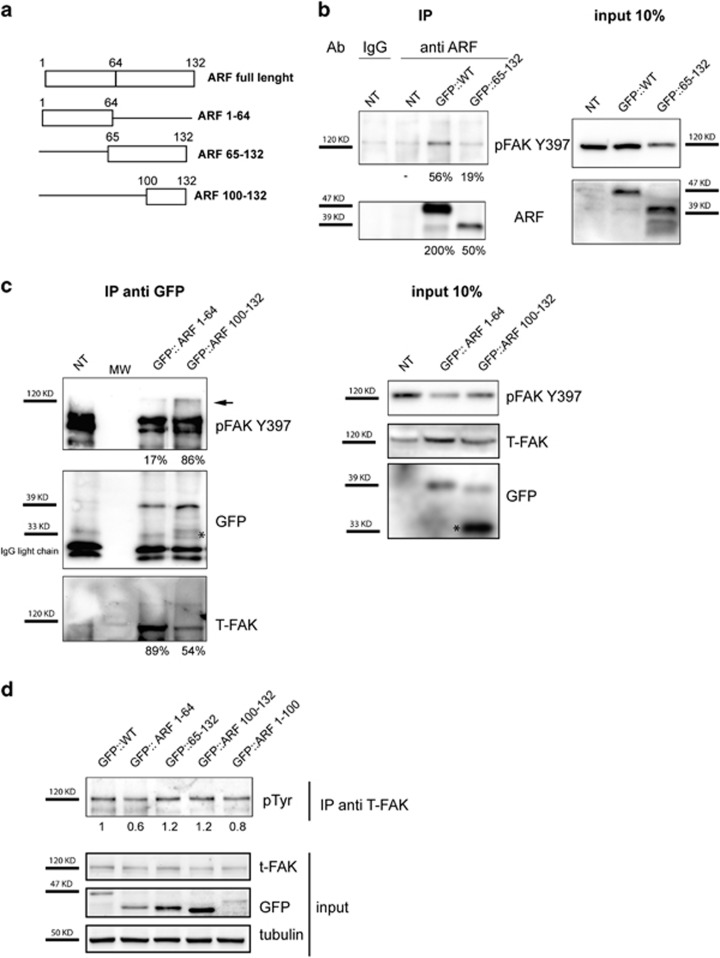
(**a**) Scheme of N-ter EGFP tagged ARF constructs used in this study. (**b**) HeLa cells were transiently transfected with wt and ARF 65–132 mutants, and after 24 h cells were re-plated and cytoplasmic extracts immunoprecipitated with anti-ARF antibody and analyzed by western blot with anti-pFAK Y397 and ARF antibodies. Panels of input are also shown on the right of immunoprecipitation experiment. Bands corresponding to the immunoprecipitated protein were quantified with ImageJ and reported as percentage of input, quantified in the same manner in order to obtain co-IP efficiency, indicated below the corresponding panel. (**c**) Cytoplasmic extracts of cells transfected with empty vector, ARF 1–64 and ARF 100–132 were immunoprecipitated with anti-GFP antibody analyzed by western blot with anti-pFAK Y397, t-FAK and GFP antibodies. Asterisk (*) indicates unspecific/degradation GFP products, arrow indicates immunoprecipitated FAK. Panel of input is also shown on the right of immunoprecipitation experiment. Efficiency of co-IP is quantified as described in **b**. (**d**) Cytoplasmic extracts were immunoprecipitated with anti-FAK antibody and analyzed by western blot with anti-pTyr antibody. Band intensity was compared with wt ARF-transfected sample arbitrarily set to 1. Panels of input probed with anti-FAK, GFP and tubulin antibodies are also shown.

In order to map ARF domain required for ARF/FAK interaction during spreading, either wt or ARF 65–132 deletion mutant were transfected in HeLa cells and subjected to immunoprecipitation (Figures 7a and b) with anti-ARF antibody. The 65–132 mutant shows reduced pFAK binding (Figure 7b) thus suggesting that a FAK-binding domain is present in the 1–64 amino-acid region. We next analyzed binding efficiency of ARF 1–64 and of ARF 100–132 by immunoprecipitation with anti-GFP. Although ARF 100–132 is still able to bind pFAK, the reduced binding shown by ARF 1–64 could reflect its impairment to induce FAK stabilization. We thus probed the filter with antibody recognizing total FAK, thus demonstrating that ARF 1–64 is still able to bind the FAK although in a not phosphorylated form (Figure 7c).

We next analyzed the ability of these mutants to stabilize FAK, by transfecting them in HeLa cells and evaluating pFAK levels during spreading. We included in this analysis an ARF deletion mutant devoid of 100–132 domain. The experiment shows that only ARF 65–132 and ARF 100–132 are able to induce FAK stabilization with an efficiency similar to the wt (Figure 7d).

Finally, we tested different ARF point mutants derived from melanoma predisposing mutations^25^ affecting single residues within an acidic motif (65–70 aa). We found that all of them are still able to induce pFAK stabilization, at efficiency similar or higher than the wt protein (Supplementary Figure S7). These experiments collectively show that ARF binding to FAK is mediated by at least two domains present in both the N-terminal and C-terminal protein region. Moreover, binding is not sufficient to induce FAK stabilization and this function is restricted to the 100–132 amino-acid region of p14ARF.

### ARF depletion induces anoikis in HeLa and HaCaT cells

**Figure 8:**
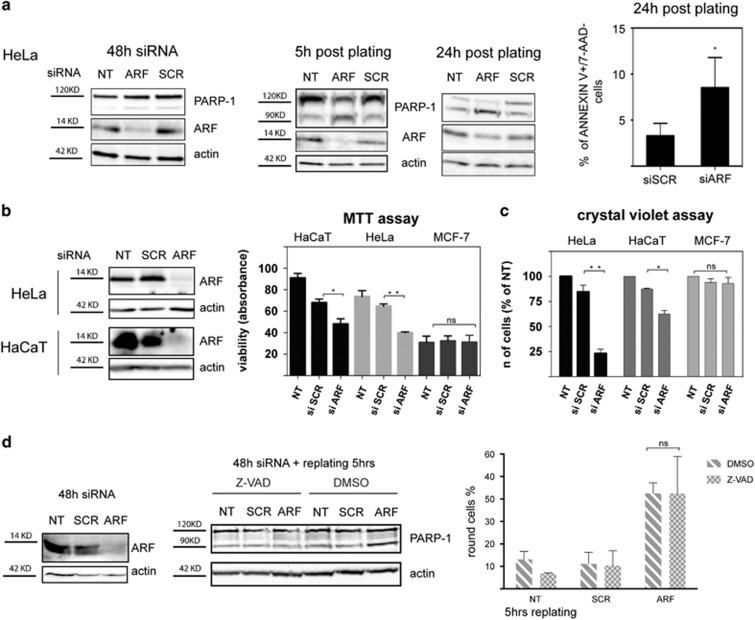
ARF depletion induces apoptosis. (**a**) HeLa cells were treated with the indicated siRNAs and analyzed by western blot 48 h later and 5 h and 24 h after plating. Silencing efficiency and viability was analyzed by western blot with anti-ARF and anti-PARP-1 antibody. Actin is a loading control. The percentage of early apoptotic cells was quantified by FACS through PE Annexin V staining. Percentage of PE Annexin V+/ 7-aminoactinomycin D-negative cells in siSCR and siARF-treated cells was reported in the graph. Cumulative data are expressed as a mean value±s.d. of three independent experiments. Asterisk indicates statistically significant differences by paired two-tailed Student's *t*-test with *P*<0.05. (**b**) HeLa, HaCaT and as negative control, MCF-7 cells (bearing INK4a/ARF locus deletion) cells were transiently transfected with the indicated siRNAs for 72 h. Silencing efficiency was analyzed by western blot with anti-ARF antibody. Actin is a loading control, whereas cell viability was analyzed by a MTT (3-(4,5-dimethylthiazol-2-yl)-2,5-diphenyltetrazolium bromide) based assay. The absorbance of each well (NT, siSCR and siARF) is reported. Graphs represent mean values±s.d. of three independent experiments. Asterisks (*) indicate statistically significant differences with *P*-value obtained through the RM one-way ANOVA with the Greenhouse-Geisser correction *P =0.0158, **P=0.0025. No statistical (NS) difference between samples are indicated. (**c**) HeLa, HaCaT and MCF-7 were transiently transfected with the indicated siRNAs for 5 days, and cell viability analyzed by crystal violet staining of the plates (CVS assay). The absorbance of each well (NT, siSCR and siARF) was calculated as percentage of the control wells (NT) set to 100%. Statistically significant differences calculated as described **P =0.0002, *P=0.0102. (**d**) HeLa cells were treated with control or specific ARF siRNA for 48 h and then re-plated in presence of the general caspase inhibitor Z-VAD or DMSO as control for 5 h and analyzed by western blot to check silencing efficiency (left panel) and apoptosis (middle panels) as described before and phase-contrast microscopy for analysis of cell morphology. Quantification of percentage of cells with round phenotype was performed as described before. Cumulative data are expressed as a mean value±s.d. of three independent experiments. For each experiment, 100 cells were analyzed for each transfection point. Unpaired two-tailed *t*-test shows NS difference between siARF DMSO and siARF Z-VAD-treated samples.

We next analyzed the relationship between cellular morphology and viability of ARF-depleted HeLa cells. Apoptosis analysis, by both western blot of PARP-1 cleavage and FACS-based Annexin V/7-aminoactinomycin D (7-AAD) assay, shows that ARF silencing induces 50% increase of the number of cells in apoptosis, as soon as cells were induced to spread (Figure 8a). As round cells could be easily separated from attached cells, we analyzed them separately. The experiment shows that round cells are 7-aminoactinomycin D/Annexin V-positive and showed increased PARP-1 cleavage (Supplementary Figures S8c and d).

MTT (3-(4,5-dimethylthiazol-2-yl)-2,5-diphenyltetrazolium bromide) assay and crystal violet staining show that ARF silencing significantly reduced HeLa and HaCaT cell viability (Figures 8b and c).

**Figure 9:**
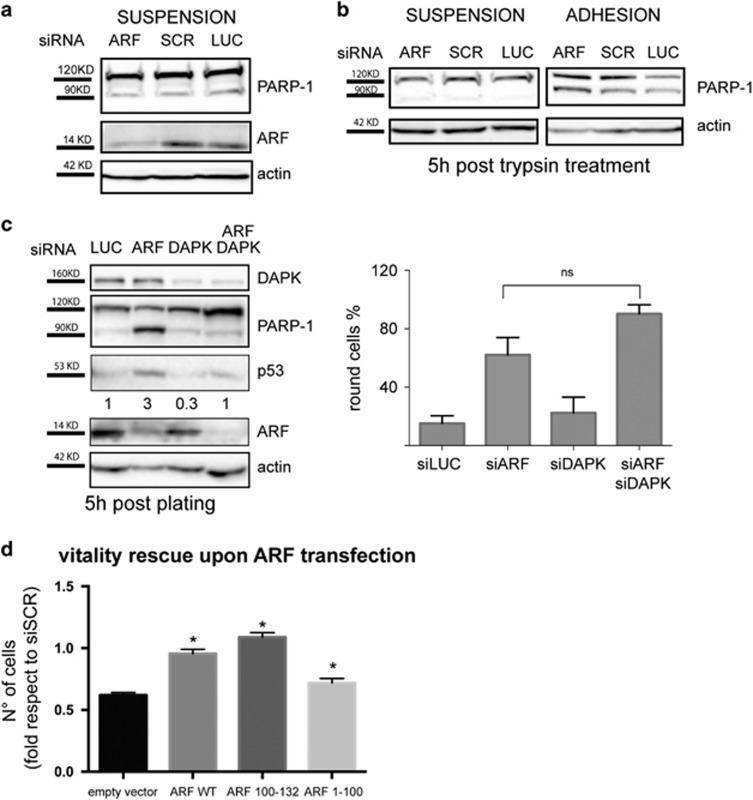
ARF depletion induces anoikis through a DAPK-dependent mechanism. (**a**) Western blot analysis of HeLa cells allowed to grow in suspension as spheres for 24 h and treated with control and specific ARF siRNA for 48 h. (**b**) Spheres were disaggregated by gentle trypsin treatment and re-placed in the same culture condition (**b**, left panels) or in adhesion (**b**, right panels) for 5 h. (**c**) HeLa cells grown in adherent conditions were treated with control or ARF and DAPK-specific siRNA alone or simultaneously for 72 h. Cells were re-plated as already described and analyzed by western blot analysis and phase-contrast microscopy. Silencing efficiency and vitality was analyzed by western blot with anti-ARF and anti-PARP-1 antibody. Actin is a loading control. WB are representative of at least three independent experiments. Normalized p53 band intensities are shown below respective wb panel, and are expressed as fold enrichment with respect to siSCR sample arbitrarily set to 1 (see Materials and methods section for details). Round phenotype was quantified and reported in graph as described. Cumulative data are expressed as a mean value±s.d. of three independent experiments. For each experiment, 100–150 cells were analyzed for each transfection point. Analysis of variance show no statistical differences between the indicated values (NS) by paired two-tailed Wilcoxon test *P*= 0.2. (**d**) HeLa cells were transiently transfected with ARF of mutant vector as indicated for 24 h and were treated with control or ARF-3 siRNA (siUTR) for 72 h. Cell viability was analyzed by CVS (cristal violet staining) assay. The absorbance of each well treated with siARF-3 was calculated as fold of the control wells (siSCR) set to 1. Graphs represent mean values±s.d. of two independent experiments. Analysis of variance shows statistical significant difference between empty vector and wt/mutant ARF-transfected points with *P*-value<0.001 by Kruskal–Wallis test.

To determine if apoptosis could be the cause of the so far described spreading defect, ARF knockdown cells were treated with the pan-caspase inhibitor Z-VAD-fmk. Quantification of spreading efficiency showed that apoptosis inhibition does not rescue the morphology defect of ARF silenced cells (Figure 8d). This could suggest that apoptosis could be induced in cells that cannot properly spread. To explore this hypothesis, we analyzed apoptosis in ARF-depleted HeLa cells grown in suspension as spheroids by changing the coating vessel surface. After 48 h of suspension culture, no PARP-1 cleavage was observed in control and ARF-depleted cells (Figure 9a) even if spheroids were disaggregated and cells re-placed under suspension culture conditions for additional 24 h (Figure 9b, left panel). Conversely, re-plating onto an adhesive substrate resulted in PARP-1 cleavage (right panel) thus indicating that cell-substrate contacts are required for apoptosis induction in cells devoid of ARF expression.

The DAPK, a pro-apoptotic serine/threonine kinase, is activated and induces apoptosis following cytoskeleton–matrix disengagement.^26, 27^ We thus investigated a possible involvement of DAPK in apoptosis induced by ARF silencing. Simultaneous abrogation of ARF and DAPK in HeLa and HaCaT cells by RNA interference rescues apoptosis induced by ARF depletion (Figure 9c; Supplementary Figure S9a) suggesting that ARF depletion induces anoikis through a DAPK-dependent mechanism. Moreover, both cell round phenotype and the decrease of pFAK levels of ARF silenced cells were not affected by DAPK abrogation, suggesting that were not caused by apoptosis (Figure 9c, graph image and Supplementary Figure S9b). Interestingly, upon ARF silencing a slight but reproducible increase of p53 levels occurred both in HeLa, where the p53 pathway is not irreversibly disabled,^28^ and HaCaT cells (Figure 9c and Supplementary Figure S9) and is reversed by concomitant DAPK depletion.

Finally, rescue experiments with wt and mutant ARF expression showed that both wt and ARF 100–132 are able to rescue cell viability (Figure 9d) thus establishing a positive correlation between increased pFAK levels and proliferation.

### ARF depletion induces actin cytoskeleton defects but not anoikis in H1299 cells

**Figure 10:**
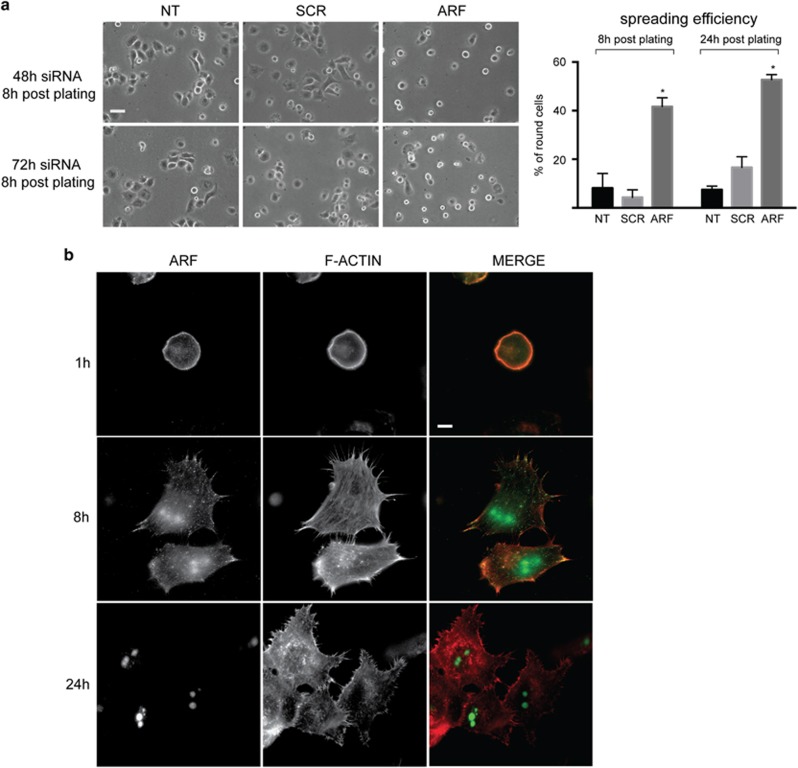
ARF is required for spreading and localizes to the plasma membrane of H1299 cells. (**a**) Contrast images of H1299 cells transiently transfected with the indicated siRNAs for 48 h and 72 h treated as described in Figure 4 and analyzed by phase-contrast microscope 8 h after plating, (scale bar, 30 μm). Quantification of round phenotype was performed as described and is shown in the graph on the right. Cumulative data are expressed as a mean value±s.e.m. of at least three independent experiments. For each experiment, 100–150 cells were analyzed for each transfection point. Asterisks indicate statistically significant differences between ARF and SCR treated cells with *P-*value obtained by unpaired two-tailed *t*-test<0.05. (**b**) H1299 cells were detached, re-suspended in growth medium, allowed to adhere onto coverslips and were fixed at different time points after plating (1, 8 and 24 h). Permeabilized cells were subjected to IF with anti-ARF antibody and stained with tetramethylrhodamine phalloidin to visualize actin cytoskeleton. Representative images showing ARF subcellular localization are shown for each time point. Images were taken by Nikon fluorescence microscopy (scale bar, 5μm).

To get further inside on the role of ARF in cell spreading and FAK phosphorylation, we extended our analysis to the H1299 cells, expressing robust endogenous levels of p14ARF. Upon ARF silencing, almost 50% cells failed to properly spread upon re-plating until 24h after seeding (Figure 10a). Analysis of ARF localization during adhesion and spreading by IF confirmed ARF recruitment at points of active actin polymerization in a time-dependent manner (Figure 10b, 8 h after plating).

**Figure 11:**
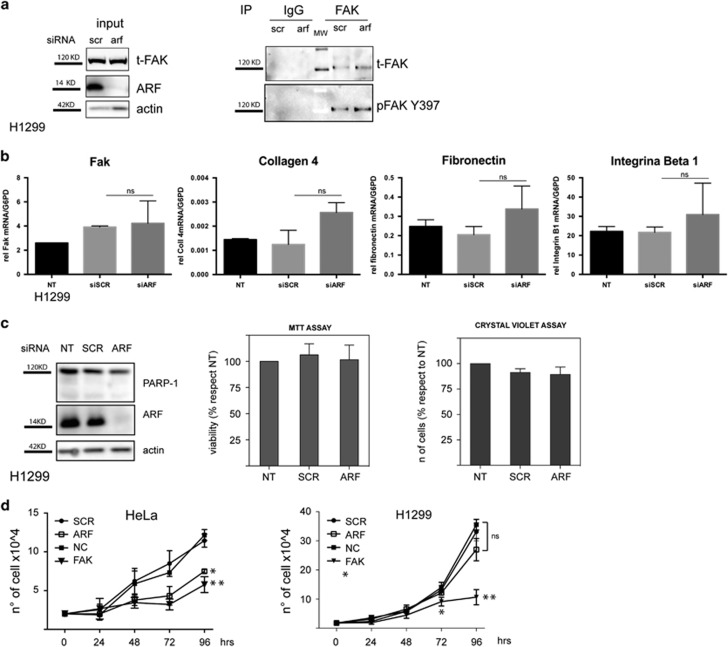
FAK levels and cell viability are unaffected by ARF depletion. (**a**) H1299 cells treated with control SCR and ARF-specific siRNA were detached and induced to adhere. Total cell extracts (8 h after re-plating) were immunoprecipitated with anti-FAK antibody or with IgG as negative control and analyzed by western blot with pFAK Y397 antibody and subsequently with anti-t-FAK antibody. Panels of inputs, representative of three independent experiments, probed with anti-FAK, ARF and actin antibodies are also shown. (**b**) Quantitative reverse transcriptase (qRT)–PCR analyses of FAK, collagen IV, fibronectin and integrin β1 mRNA levels normalized on G6PD expression in cells treated with the indicated siRNA are shown. Cumulative data are expressed as a mean value±s.d. of three independent experiments. Statistical analysis has been performed as described in Figure 4. Not statistically significant differences are indicated (NS). (**c**) H1299 cells were transiently transfected with the indicated siRNAs and silencing efficiency analyzed by western blot with anti-ARF and actin as loading control. Viability was checked with PARP-1 antibody, MTT (3-(4,5-dimethylthiazol-2-yl)-2,5-diphenyltetrazolium bromide) and CVS (cristal violet staining) assay as described previously. Data are expressed in **c** and **d** are mean value±s.d. of three independent experiments. (**d**) Growth curve of HeLa and H1299 cells treated with the indicated siRNA at different time points. Asterisks indicate statistically significant differences by unpaired two-tailed *t*-test, between FAK and NC sirna treated cells **P=0.0048, or between ARF and SCR sirna treated cells *P=0.0085 for HeLa cells. In H1299 cells, *P=0.0217 between FAK and NC at 72 h and **P= 0.0002 at 96 h. Not statistically significant differences are indicated (NS).

Interestingly, we found that, in contrast with what described previously, FAK levels do not change upon ARF silencing (Figure 11a). In line with this, no difference in FAK RNA levels nor in other adhesion-related genes were found between ARF and control-depleted cells (Figure 11b). These results suggest that the spreading defect does not depend on FAK levels in these experimental conditions. Consistently, transient FAK silencing in HeLa cells does not completely phenocopy ARF depletion as cells retain the ability to spread (Supplementary Figure S10a). These experiments collectively show that in H1299 cells, the molecular circuitry linking cytoskeleton dynamics and FAK levels are disconnected, thus implying that the round phenotype relies on specific ARF-dependent functions.

Interestingly, when we analyzed the role of ARF on cell viability in H1299, we found no effect on cell growth (Figure 11c). Moreover, cellular growth curves showed that, although ARF is required for proliferation of HeLa and HaCaT cells but it is dispensable for H1299, all tested cell lines require FAK expression to proliferate (Figure 11d and Supplementary Figure S10b). In agreement, western blot analysis shows that FAK-depleted HeLa and H1299 cells present PARP-1 cleavage and thus undergo apoptosis (Supplementary Figure S10a and data not shown), suggesting that, rather than having a role in spreading, FAK downregulation is the signal that halts cell growth in these cell lines.

**Figure 12:**
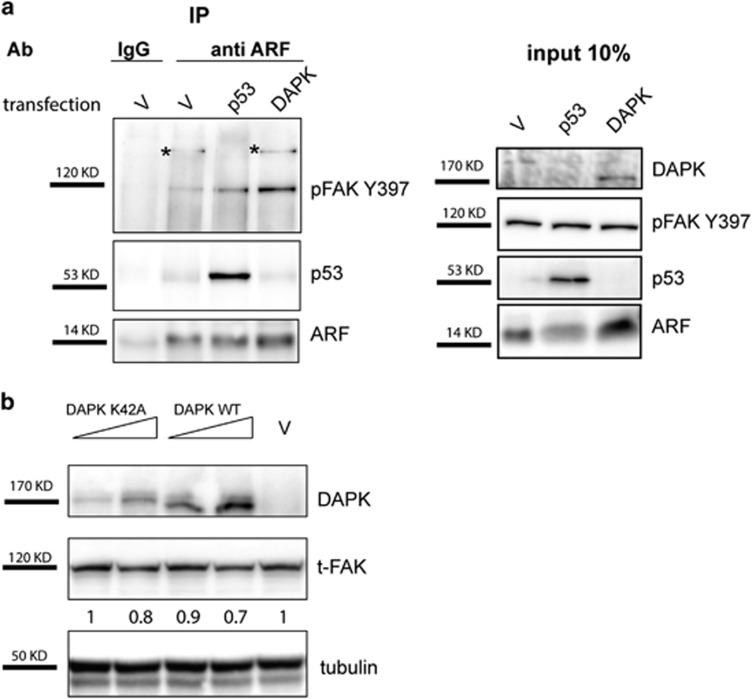
ARF interacts with FAK in H1299 cells. (**a**) H1299 cells were transiently transfected with empty vector (v) or either p53 or DAPK-expressing plasmids. After 24 h, cells were re-plated and cytoplasmic extracts immunoprecipitated with anti-ARF antibody and analyzed by western blot with anti-pFAK Y397, ARF and p53 antibodies. Panels of input are also shown on the right of immunoprecipitation experiment. (**b**) H1299 cells were transfected with an empty vector (v) or increasing amount of wt DAPK or DAPK K42A expression plasmids. Twenty-four hours after transfection, cells were lysed and cellular extracts were immunoblotted and analyzed with anti-FAK, anti-DAPK and anti-tubulin as loading control. WB shown are representative of at least three independent experiments. Normalized FAK band intensities are shown below each corresponding band, and are expressed as fold enrichment with respect to empty vector-transfected sample arbitrarily set to 1.

**Figure 13:**
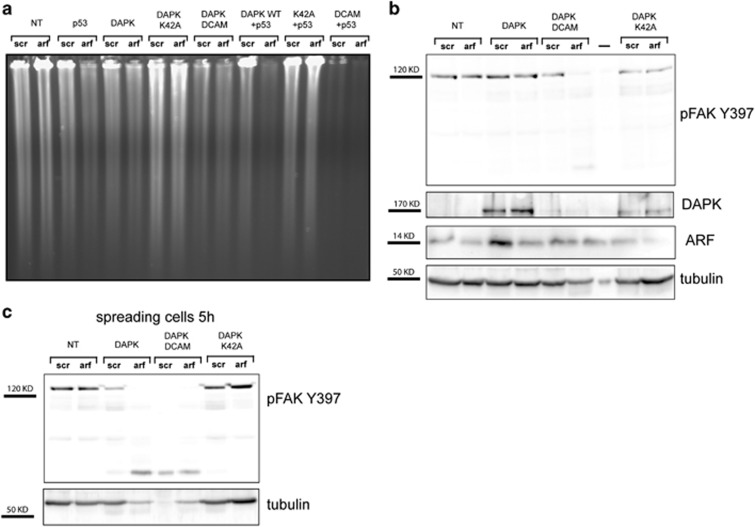
DAPK re-introduction in H1299 cells induces apoptosis and decrease of pFAK levels upon ARF silencing. (**a**) Genomic DNA content analysis for detection of apoptosis. H1299 cells were transiently transfected with the indicated vectors for 24 h and treated with siSCR control or ARF siRNA for 48 h. Genomic DNA was extracted and analyzed by agarose gel electrophoresis to analyze DNA integrity. (**b**) Western blot analysis showing pFAK Y397 levels upon transfection and silencing in the same experimental conditions. Tubulin was used as loading control. (**c**) Same experiment shown in **b** but collected 5 h upon spreading induction.

We thus tested ARF ability to interact with FAK in this cell line. Moreover, as neither p53 nor DAPK are expressed in these cells, we tested if their exogenous expression could have a role in this interaction. The experiment shows that ARF and FAK interact in this cell line and the binding increases when DAPK is transfected (Figure 12a). Interestingly, transient transfection of both wt and DAPK K42A, a mutant defective in apoptosis induction, induce a decrease in FAK levels (Figure 12b). We thus wondered if it could be possible to induce apoptosis and FAK decrease upon ARF silencing in H1299 cells following DAPK and p53 re-introduction by transient transfection. As a key feature of apoptotic cell death is genomic DNA fragmentation, we extracted genomic DNA and analyzed its integrity by agarose gel electrophoresis. As expected, ARF silencing does not affect genomic DNA integrity in this cell line (Figure 13a). Transfection of p53 and wt DAPK slightly affect genomic DNA integrity, whereas DAPK K42A is not functional. Interestingly, ARF silencing further induces degradation of genomic DNA in cells expressing either p53 or WT DAPK, even more when both proteins are expressed, whereas it does not in cells expressing the inactive DAPK K42A mutant. Cells transfected with the DAPK constitutive active mutant (DAPK DCAM) strongly show DNA degradation irrespective of ARF status. Analysis of pFAK levels shows that ARF silencing has no effect of pFAK levels in untransfected cells (Figures 13a and b). DAPK expression instead induces a decrease of FAK expression when ARF is silenced (Figure 13b, compare lane 4 with lane 3). Expression of constitutive active DAPK DCAM strongly induces apoptosis and decrease of FAK levels. This effect is further exacerbated when cells are induced to spread (Figure 13c).

## Discussion

Here we show, for the first time, that although in adherent cells ARF localizes mainly in the nucleus, during the early phase of the spreading process the protein is recruited or stabilized to cell periphery at points of focal adhesions, in a precise time window. This localization is substrate independent, and is involved in the organization of actin structures during cell spreading. Reducing ARF expression levels leads to defects in the spatial organization of actin cytoskeleton during the process of cellular spreading. In line with a role in this phenomenon, we noted that during cell spreading ARF protein levels increase, through a protein kinase C-dependent mechanism (Fontana, personal communication). Interestingly, it has been reported that p19ARF-deficient MEFs display a round phenotype, similar to the one reported in our study, accompanied by activation of the PI3K-Rac1 pathway and increased migration properties.^29, 30^ Despite significant divergence in amino-acid sequences, we show that this function is evolutionary conserved and the expression of p19 *per se* increases spreading efficiency (Figure 5c). In addition, our preliminary experiments show that, in line with the aforementioned article, ARF-depleted cells acquire increased migration properties.

Cell adhesion to the substrate is primarily mediated by integrins that, connecting extracellular matrix with cytoskeleton, induce cell survival pathways including phosphorylation and activation of FAK,^31, 32^ whose activation strongly correlates with tumor aggressiveness, cell migration and proliferation.^33, 34, 35^ Here we provide evidence that p14ARF physically interacts with the FAK in its activated form. Given the observed reduction of total and phosphorylated FAK during the cell spreading process in ARF-depleted cells, we postulate that, through physical interaction, ARF can exert a protective role on FAK upon detachment and adhesion. Our experiments show that although the domains necessary for FAK interaction are present both in the N-ter and C-ter portion of the protein, the domain required for pFAK stabilization is restricted to the exon 2 encoded region. It has to be underlined that ARF region 100–132, required for both pFAK activation and survival, has an established role in autophagy as reported in Budina-Kolomets *et al.*^36^ thus suggesting that ARF ability to regulate autophagy could be involved in this mechanism and it is independent from the reported ARF functions in mitochondrial homeostasis.^25^ However, from experiments performed with mitotracker staining, we cannot exclude that ARF localization to cell periphery during the early phases of cell spreading (data not shown) could be in part mediated or primed by mitochondria accumulation to sites of actin remodeling.

It has been reported that sumoylated FAK^20^ shows increased stability compared with the un-modified counterpart (Kadare *et al.*^20^ and our unpublished data), whereas ARF has been shown to mediate sumoylation of its interacting partners.^21, 37^ Our data show that ARF expression induces the appearance of slow migrating FAK species, thus supporting the hypothesis that by interacting with FAK it could mediate its sumoylation and stabilization.

Proper organization of cytoskeletal structures allows the transduction of survival pathways inside the cells whose deregulation can result in anoikis.^38, 39, 40, 41, 42^ Resistance to anoikis is a hallmark of malignant tumor cells, resulting in anchorage independence. DAPK is an actin-associated pro-apoptotic protein having an important role in tumor suppression^43, 44^ and anoikis.^26, 45^ Our data show that cell death induced by ARF depletion is DAPK dependent, as we observe anoikis rescue in doubly depleted ARF/DAPK cells. It has been previously reported that DAPK can phosphorylate ARF *in vitro* and thus promote p53-dependent apoptosis.^39^ Recently, we have shown that phosphorylated ARF species are more stable in the cytoplasm.^46^ As we noticed a decrease of ARF protein levels upon DAPK silencing (see Figure 8c), we speculate that DAPK could increase ARF stability through phosphorylation, suggesting the existence of a feedback loop between ARF and DAPK, in which ARF blocks DAPK-mediated apoptosis during cytoskeleton remodeling, whereas DAPK exerts a positive effect on ARF stability. Moreover, our data show an increase of p53 protein levels and of its transcriptional targets Puma and Apaf-1 (preliminary data not shown) upon ARF depletion, suggesting that DAPK apoptosis induction could be in part mediated through p53. Interestingly, it has been observed that ARF expression in spermatogonia prevents p53-dependent apoptosis.^47^ Further experiments are needed to validate this hypothesis.

It has been reported that overexpression of DAPK induces morphological changes typical of apoptotic cells, such as cell rounding and shrinkage.^48^ Our data only partially fit with these observations, as we do not observe a rescue of round phenotype in DAPK-depleted cells. In line with these results, we observe that H1299 cells, not expressing DAPK, show a round morphology upon ARF depletion and result in anoikis resistance.

DAPK-mediated apoptosis is accompanied by a specific decrease of FAK tyrosine phosphorylation.^45^ As in H1299 we do not observe a decreased FAK activation upon ARF silencing, we hypothesized that the FAK downregulation observed in HeLa and HaCaT cells is at least in part due to activation of DAPK upon ARF depletion. In line with this hypothesis, re-introduction of both wt and death inactive DAPK mutant, induces a strong decrease of FAK levels upon cytoskeleton remodeling. More importantly, restoration of DAPK expression in H1299 cells induces apoptosis and this effect is further amplified by concomitant ARF silencing.

**Figure 14:**
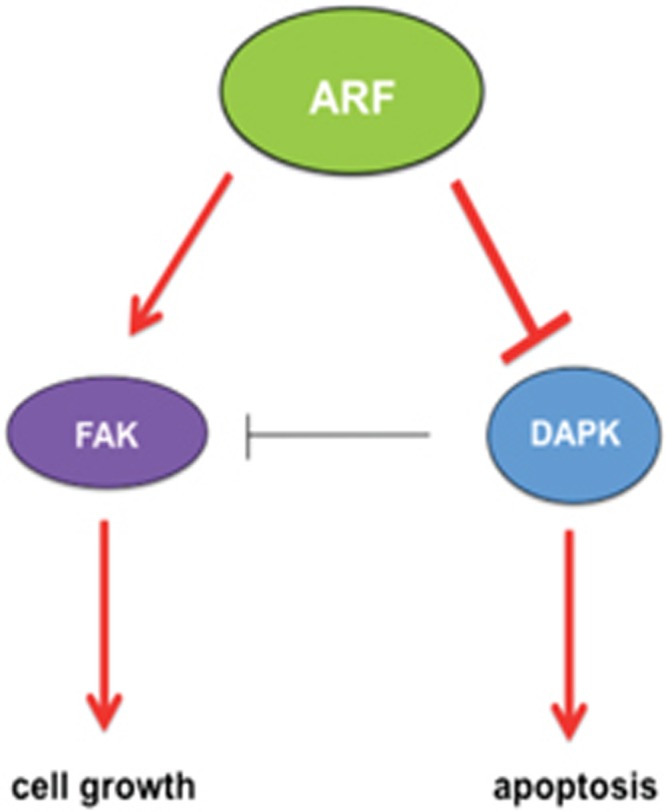
Proposed schematic model for ARF-mediated regulation of cell growth and inhibition of apoptosis. During cellular adhesion and spreading, ARF expression stabilizes activated FAK in the cytoplasm, probably mediating its sumoylation. FAK stabilization sustains cell growth. In parallel, ARF expression blocks DAPK-induced apoptosis, through both p53-dependent and -independent mechanism protecting cells from anoikis. In addition, ARF hampers DAPK downregulation of FAK activity. In cell-expressing p53, FAK stabilization can further results in FAK-mediated p53 degradation and thus inhibition of p53-dependent apoptosis.

On this basis, we propose that, far from being simply inactive to restrain cell growth, ARF confers pro-survival properties by helping cells to spread and protecting them from anoikis as illustrated in Figure 14. This is accomplished through inhibition of DAPK-induced apoptosis, and stabilization of FAK.

Conflicting data exist on ARF pro-oncogenic activity in some experimental settings. In particular, it has been reported that ARF contributes to prostate cancer progression by stabilization through SUMO modification of Slug protein.^37^ Moreover, the ability of ARF to induce autophagy, a pro-survival mechanism related to the phenomenon of tumor dormancy,^49^ also points to the hypothesis that in particular cellular contexts ARF could have a pro-proliferative or even oncogenic role. In this regard, it is interesting to note that the effect of ARF loss on cell morphology of prostate cells^37^ is the opposite of that observed in MEF cells and in our experimental conditions.

Our findings point to a novel role of ARF on protection from cell anoikis. This could have a strong impact on tumor growth and dissemination. By providing insight into these previously unknown functions of this protein, this study may have important implications in development of novel strategies for cancer therapy.

## Materials and methods

HeLa cells were obtained from Dr Valeria R Villella, San Raffaele Scientific Institute, Milan, Italy. All other cell lines (H1299, HaCaT and MCF-7) were purchased from Cell Line Service (CLS, Eppelheim, Germany). Cells were grown as described,^15^ transfected with Lipofectamine 2000, RNAiMAX reagent or electroporation (Neon transfection System, Life Technologies Carlsbad, CA, USA). Cells were routinely tested for mycoplasma contamination and kept in culture for no more than 6 weeks.

ARF siRNAs (harboring the stealth modification) anneal in the exon 1β of p14ARF transcript. ARF and p16INK4a siRNA sequences has been reported *in Vivoet al.*^8^ and Kobayashi *et al.*^50^ DAPK, FAK and luciferase siRNAs are available by Qiagen (Hilden, Germany). All siRNA were transiently transfected at a final concentration of 10 μM, except siRNA-3 that was used at 30 μM. Fibronectin purified from bovine plasma (Millipore, Billerica, MA, USA; 10 μg/ml). Z-VAD fmk (BioVision, Milpitas, CA, USA) was used at final concentration of 10 μM.

### Quantitative reverse transcriptase–PCR analysis

Equal number of cells was lysed with RNA Mini Kit Ambion (Carlsbad, CA, USA). In all, 1 μg of total RNA of each sample was reverse-transcribed and quantitative PCR amplifications were performed as described *in Vivoet al.*^8^ Primers were used: p16INK4a forward 5′-CCAACGCACCGAATAGTTACG-3′, reverse 5′-GCGCTGCCCATCATCATG-3′. FAK, DAPK, collagen IV, fibronectin and integrin β1 primers were purchased from Qiagen (Quantitect Primer Assay); G6PD forward 5′-ACAGAGTGAGCCCTTCTTCAA-3′ and reverse 5′-GGAGGCTGCATCATCGTACT-3′. Data presented represent the mean of three biological replicates.

Western blot was performed as described.^15^ Band intensities were quantified by ImageJ Software (free software, NIH), normalized to actin and reported in graph as percentage of control. Each profile represents the mean of three independent experiments.

The list of antibodies are as follows: anti-PARP-1, (Cell Signaling Denvers, MA, USA, #9542); anti-ARF C-18, anti-actin I-19, anti-p53 DO-1, anti-caspase-3 H-277, anti-FAK C-20, anti-pFAK (Tyr397)-R for IF, (Santa Cruz Biotechnology, Heidelberg, Germany), anti-pFAK (Tyr397) (BD Milan, Italy; clone 14 for wb), anti-ARF Ab2 14PO2 (Neomarkers from Bio-Optica, Milan, Italy); anti-p19ARF (Novus Biologicals from Novus Italy, Milan, Italy; Ab80) anti-DAPK-55 mouse monoclonal antibody (Sigma Aldrich, Milan, Italy); anti-pPaxillin Tyr118 (Life Technologies) and anti-pTyr 05-321 (Upstate from Millipore, Darmstadt, Germany).

#### Cell viability assays

Cell viability was determined by the crystal violet staining method^51^ or through a (3-(4,5-dimethylthiazol-2-yl)-2,5-diphenyltetrazolium bromide)) MTT based assay.^52^ Dye uptake was measured at 580 nm using a spectrophotometer. Cell viability was calculated as dye intensity and compared with untreated samples.

#### Microscopy and IF

Live phase-contrast images were acquired using a (Nikon Eclipse microscope, Tokyo, Japan). Cell spreading was quantified as described in Tamura *et al.*^53^ Fibronectin stimulation and IF were performed as described in Schlaepfer and Hunter^54^ and Vivo *et al.*^46^ Cells were incubated with Tetramethylrhodamine or fluorescein isothiocyanate-conjugated phalloidin for 30 min, followed by DAPI (Sigma-Aldrich) for 3 min and washed with PBS/0.05% Tween. Coverslip were mounted with Ibidi mounting medium (Ibidi GmbH, Martinsried, Germany).

Immunoprecipitation assays were performed either with cytoplasmic extracts or total cellular extracts (from 5 × 10^6^ cells per sample). Subcellular fractionation was done as previously described.^15^ For total cellular extracts, cells were harvested, lysed with RIPA buffer and incubated with anti-ARF antibody (C-18 Santa Cruz Biotechnology) or anti-FAK antibody.

Apoptosis was analyzed by PE Annexin V Apoptosis Detection Kit I (BD Biosciences; cat no. 559763) following the manufacturer’s instructions. DNA content analysis for detection of apoptotic cells was performed as described.^55^

#### Statistic

Data presented in this work was derived from experiments performed at least in triplicate (biological replicates), except when differently stated. The sample size of each experimental point is reported in the relative figure legend, as well as the specific statistical analysis performed. In all the experiments in which single cells were analyzed, 5–10 fields were randomly selected in the coverslip for each experimental point. Data have been analyzed to assay normal distribution with different tests, such as the D’Agostino and Pearson omnibus, the Shapiro–Wilk and the KS normality test. Analysis of data not showing a normal distribution were performed with the Wilcoxon, Mann–Whitney tests and Kruskal–Wallis test, otherwise *t*-test and analysis of variance were performed using GraphPad Prism 5.0 software (La Jolla, CA, USA).
