# Supplementary material for: p14ARF interacts with the focal adhesion kinase and protects cells from anoikis
Source: Oncogene. 2017 Apr 24;36(34):4913–28. doi: 10.1038/onc.2017.104 (PMC5582215; doi:10.1038/onc.2017.104)
Supplement: Supplementary Figure S1 [file onc2017104x1.pdf]

Fig S1

a

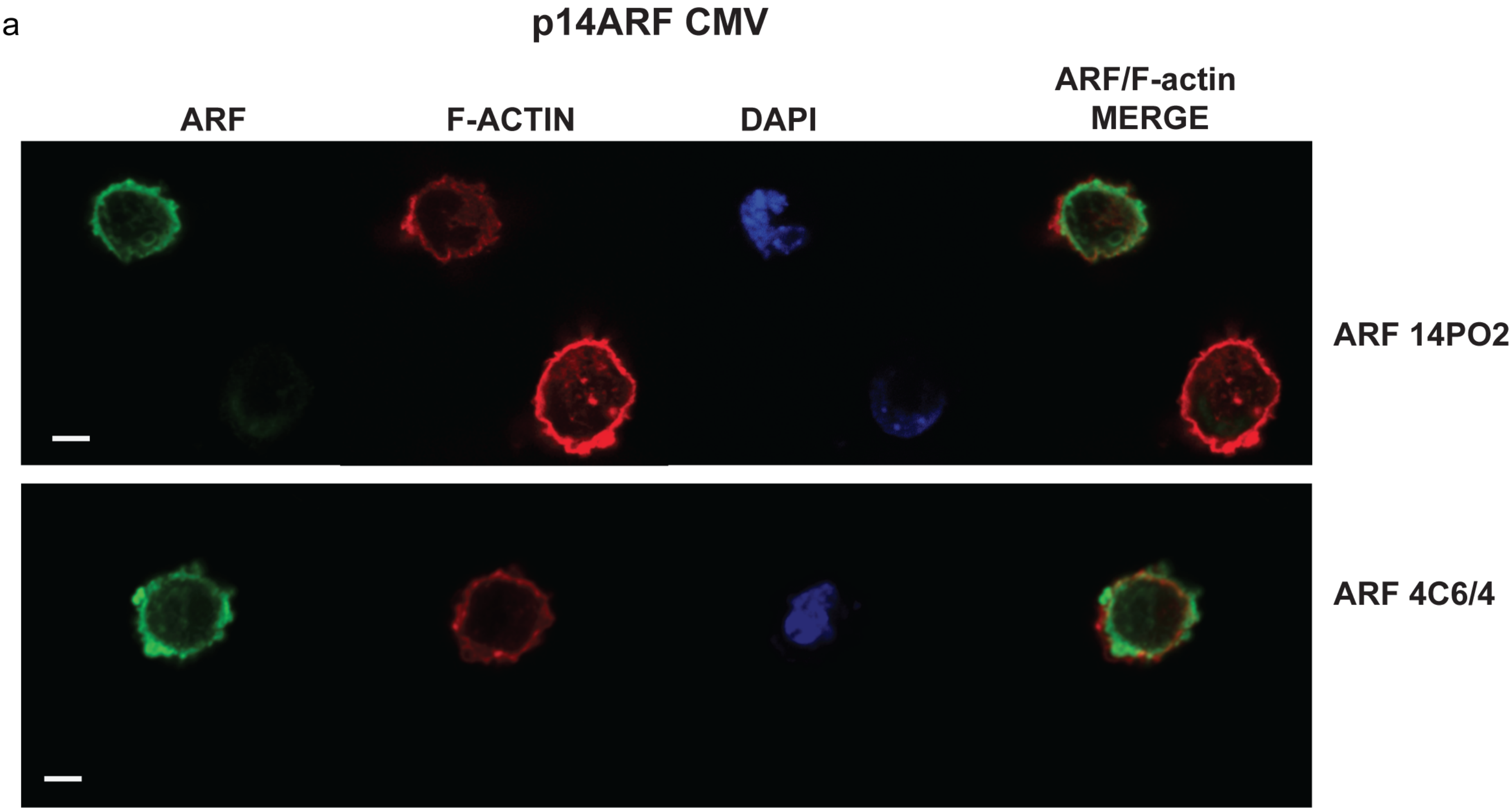
p14ARF CMV
ARF
F-ACTIN
DAPI
ARF/F-actin MERGE
ARF 14PO2
ARF 4C6/4

b

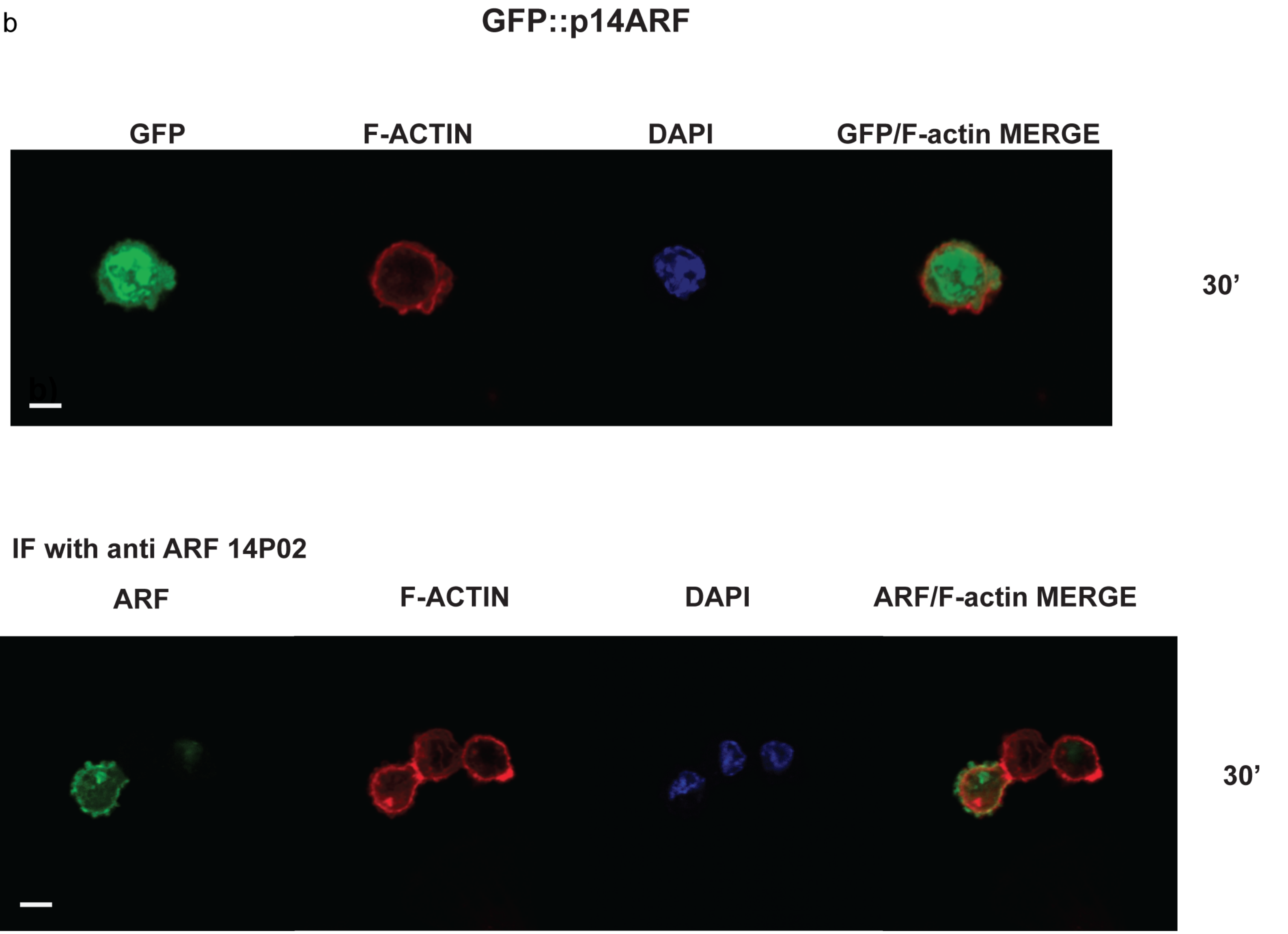
GFP::p14ARF
GFP
F-ACTIN
DAPI
GFP/F-actin MERGE
30’
IF with anti ARF 14P02
ARF
F-ACTIN
DAPI
ARF/F-actin MERGE
30’
