# Supplementary figures and images for: p14ARF interacts with the focal adhesion kinase and protects cells from anoikis
Source: Oncogene. 2017 Apr 24;36(34):4913–28. doi: 10.1038/onc.2017.104 (PMC5582215; doi:10.1038/onc.2017.104)

Fig S2

f-actin- ARF localization 3D matrigel

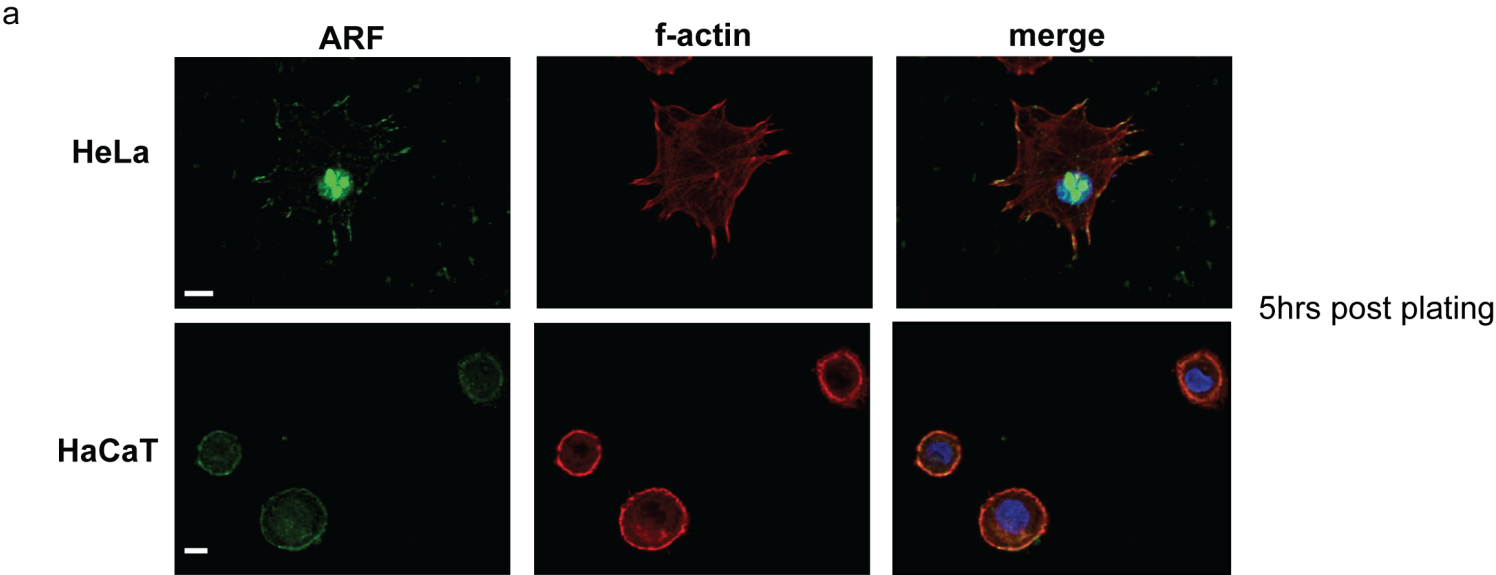

b ARF- B23 localization time course replating

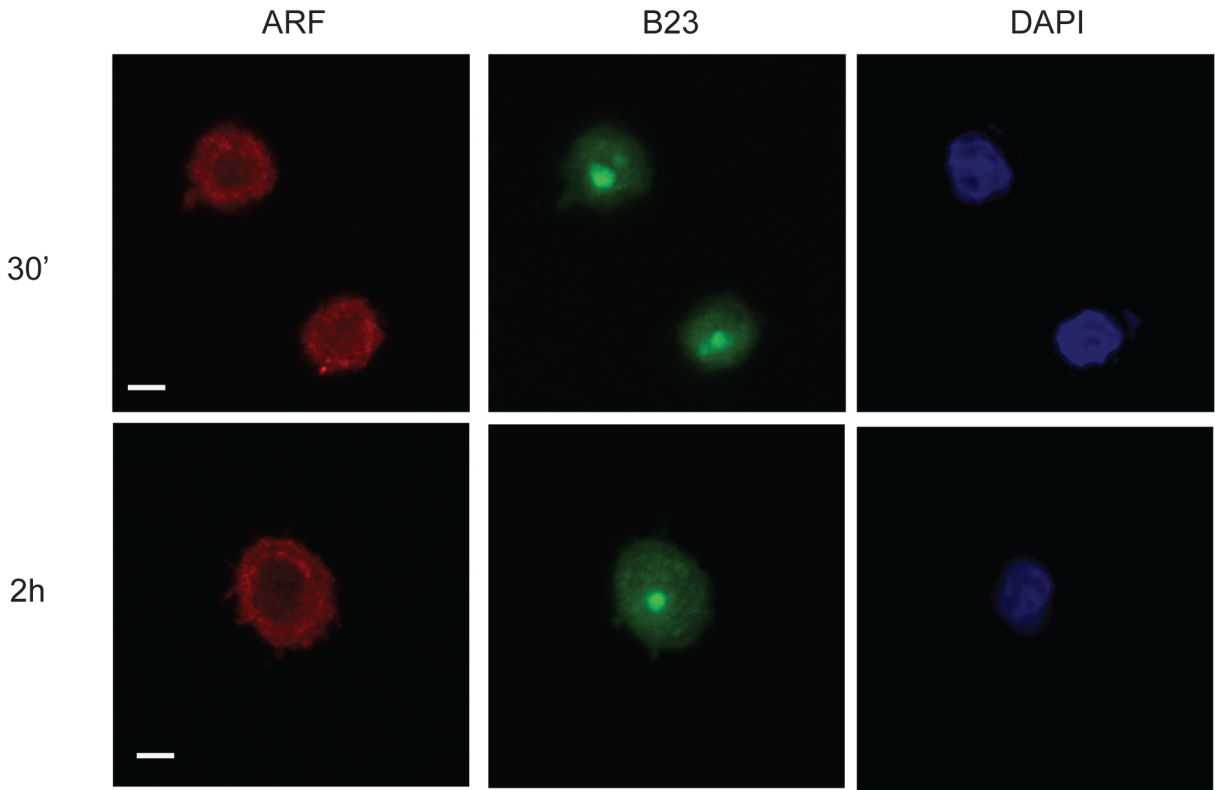

Supplement: Supplementary Figure S2 [file onc2017104x2.pdf]

**Fig S3**

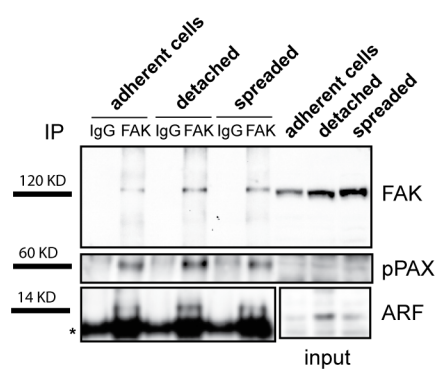

Supplement: Supplementary Figure S3 [file onc2017104x3.pdf]

Fig S5

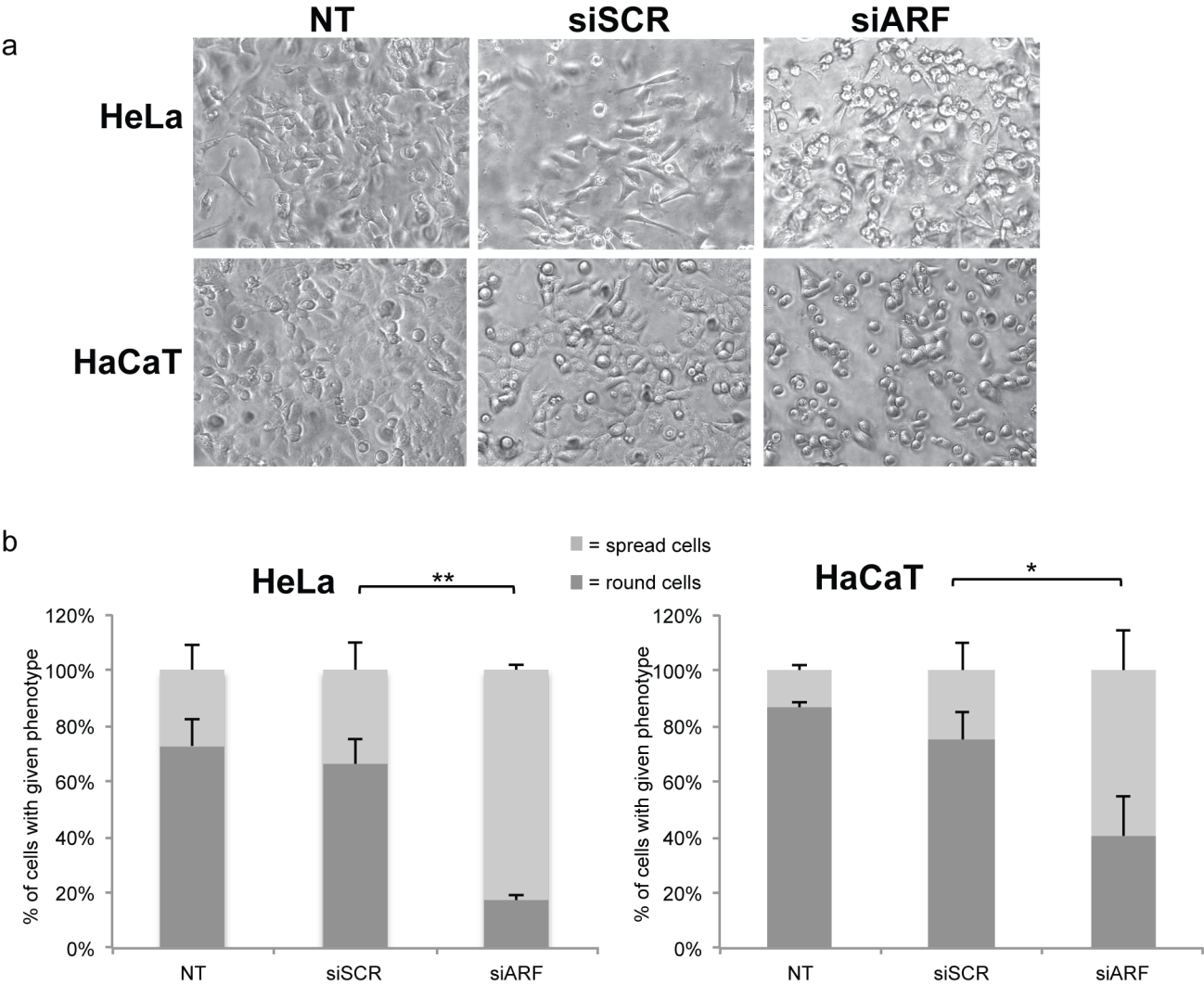

Supplement: Supplementary Figure S5 [file onc2017104x5.pdf]

Fig S6

a

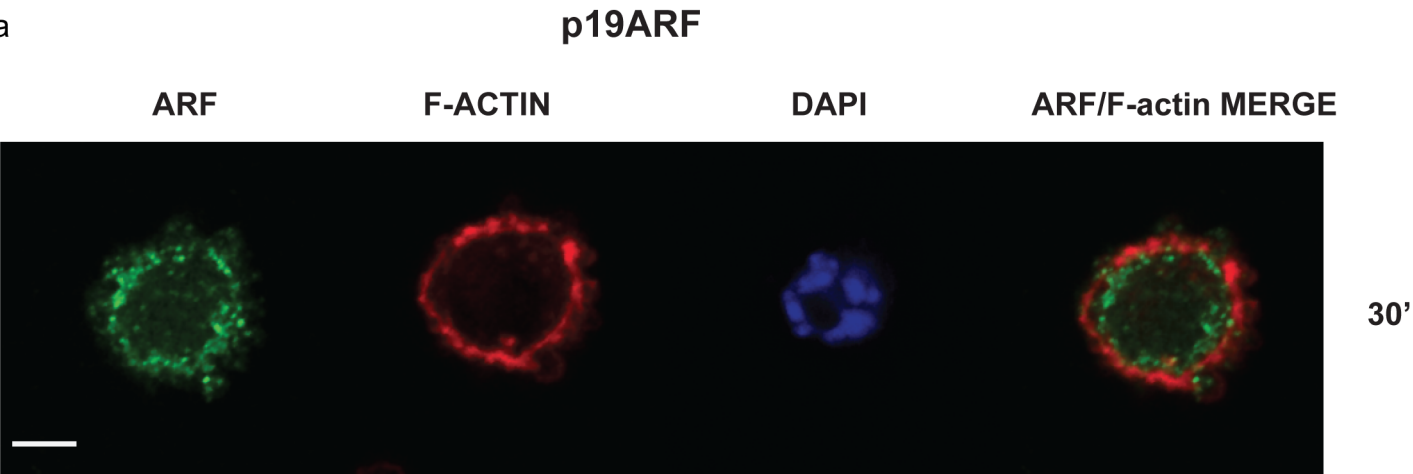

b

IP anti FAK  
WB anti pFAK Y397

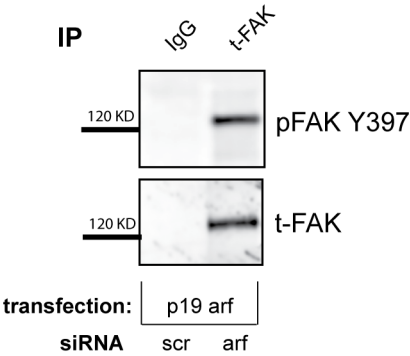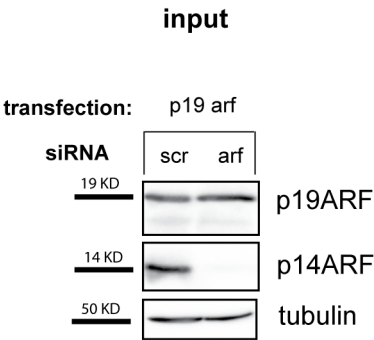

Supplement: Supplementary Figure S6 [file onc2017104x6.pdf]

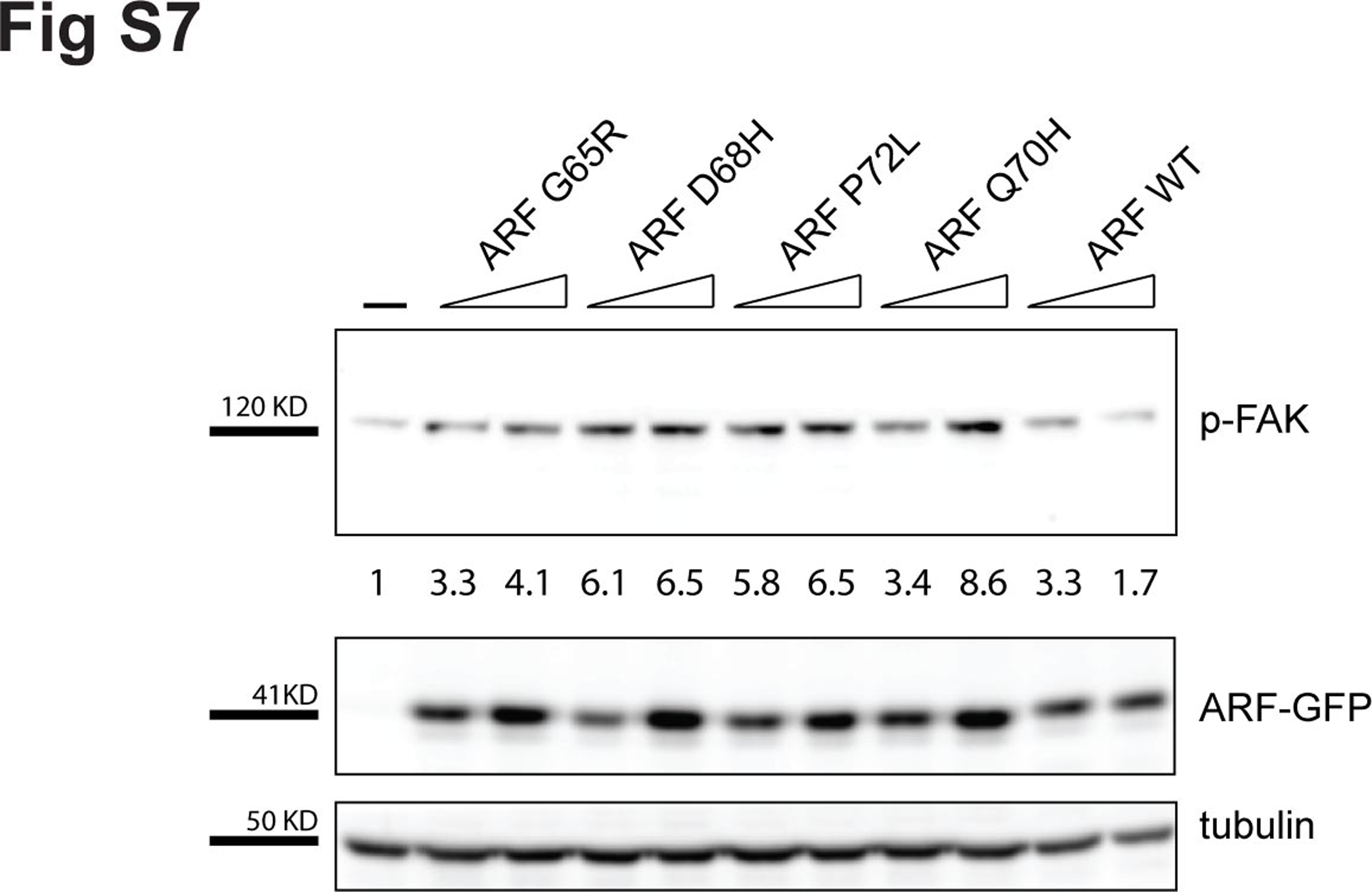

Supplement: Supplementary Figure S7 [file onc2017104x7.tif]

Fig S8

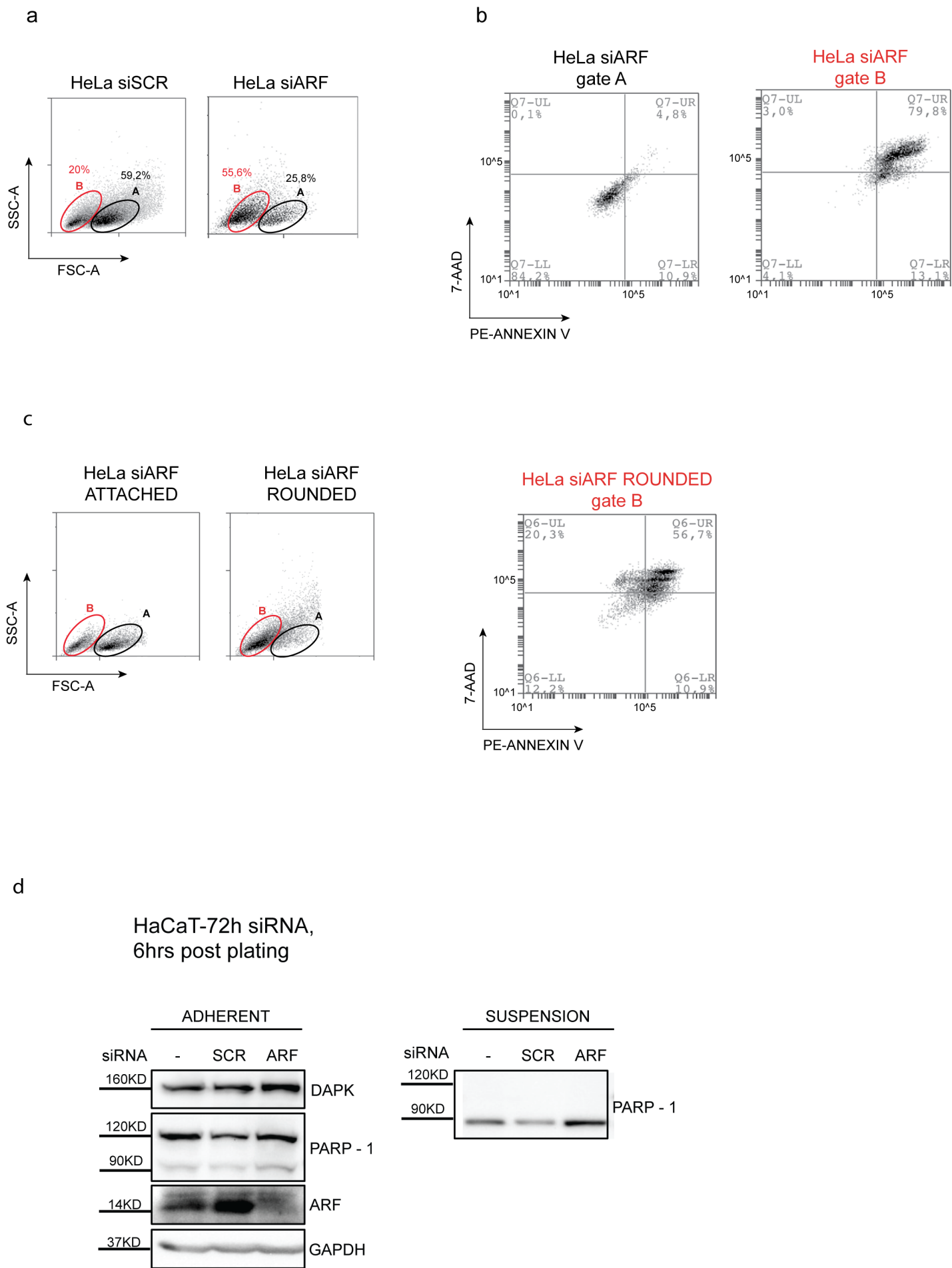

Supplement: Supplementary Figure S8 [file onc2017104x8.pdf]

Fig S9

a HaCaT cells

6h post plating

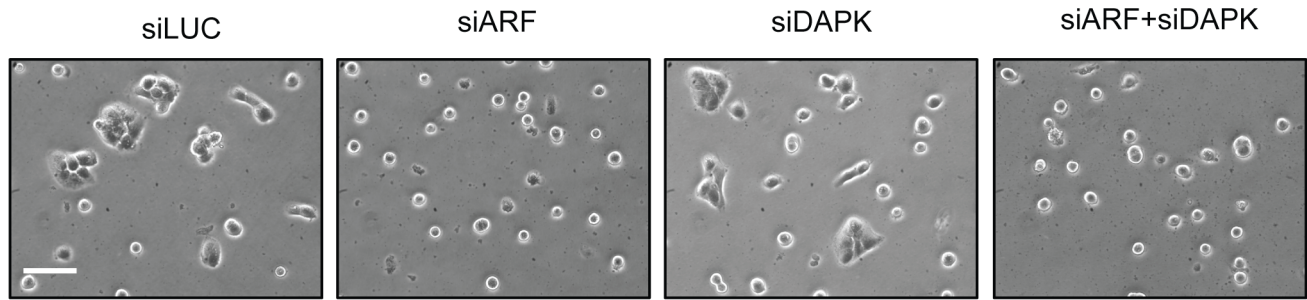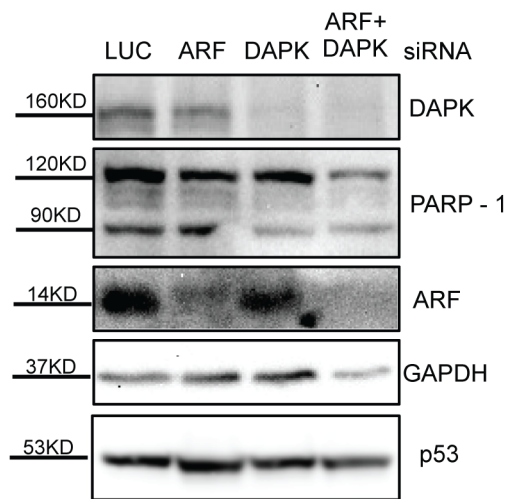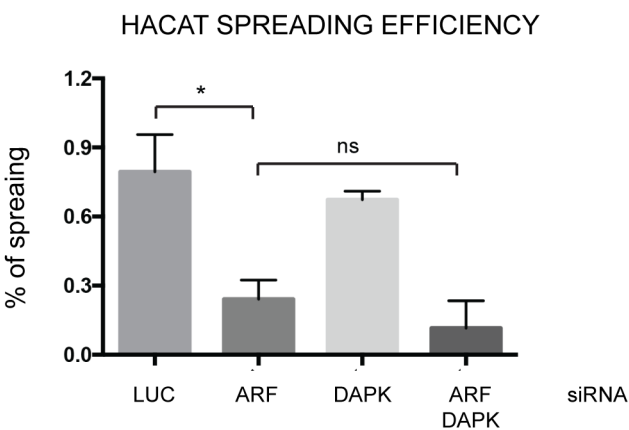

b HeLa cells 5h post plating

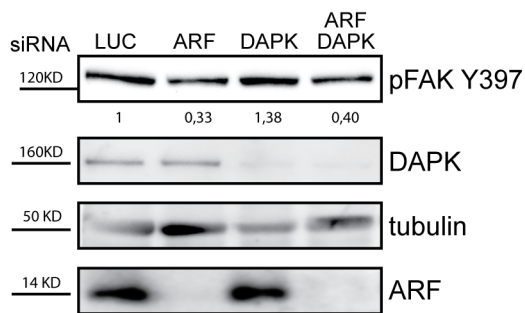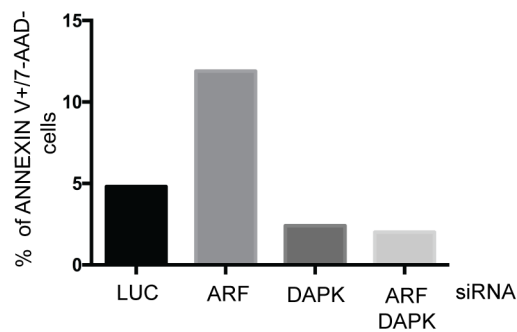

Supplement: Supplementary Figure S9 [file onc2017104x9.pdf]

Fig S10

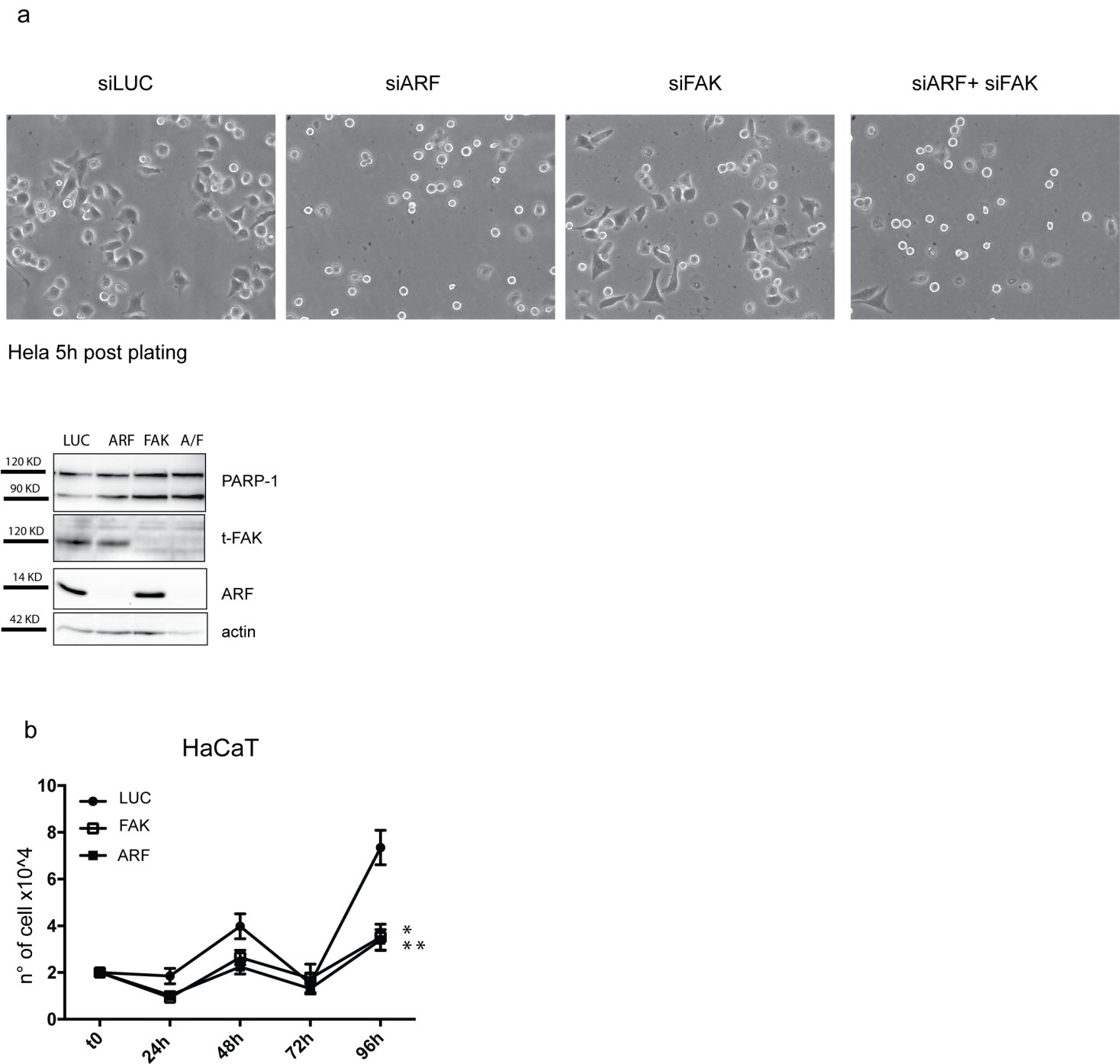

Supplement: Supplementary Figure S10 [file onc2017104x10.pdf]
