# Supplementary material for: p14ARF interacts with the focal adhesion kinase and protects cells from anoikis
Source: Oncogene. 2017 Apr 24;36(34):4913–28. doi: 10.1038/onc.2017.104 (PMC5582215; doi:10.1038/onc.2017.104)

Fig S4

EFFECT OF DIFFERENT ARF TARGETING siRNA ON p14ARF and p16INK4a LEVELS

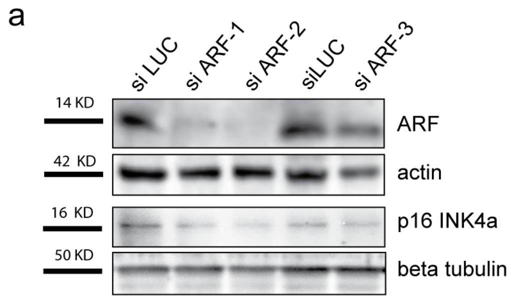

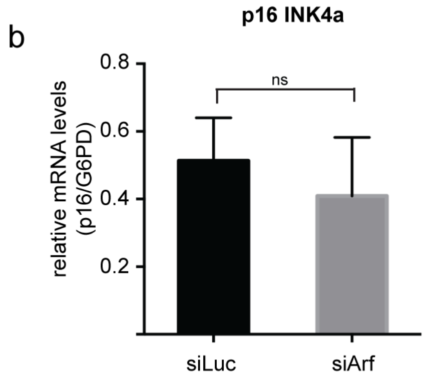

c

EFFECT OF DIFFERENT ARF siRNAs ON CELL SPREADING

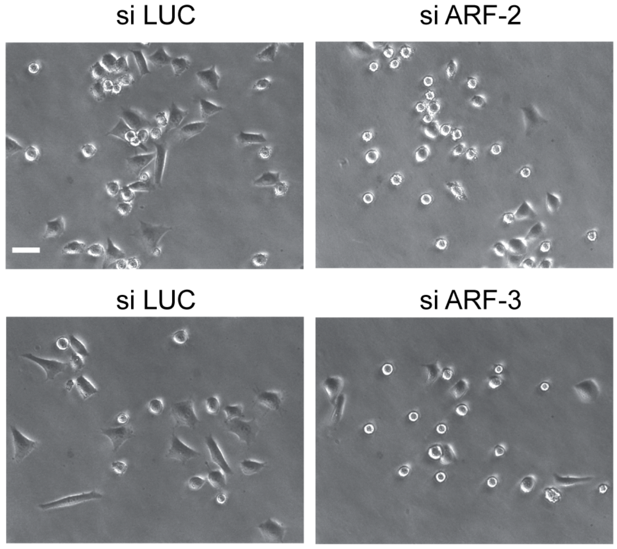

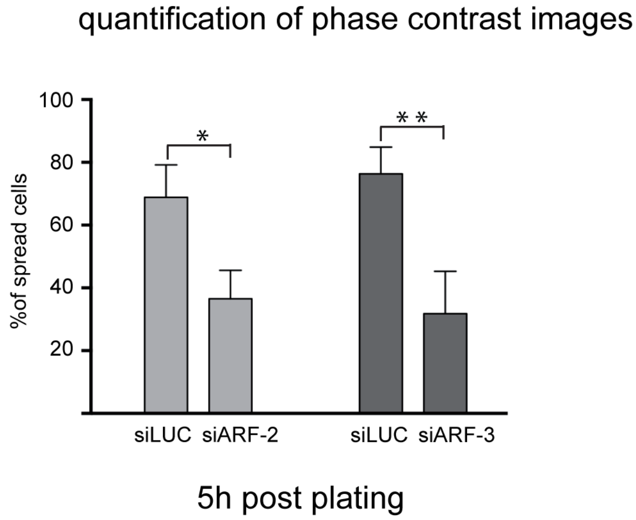

phase contrast images

d

p16 siRNA DOES NOT AFFECT HELA CELL SPREADING

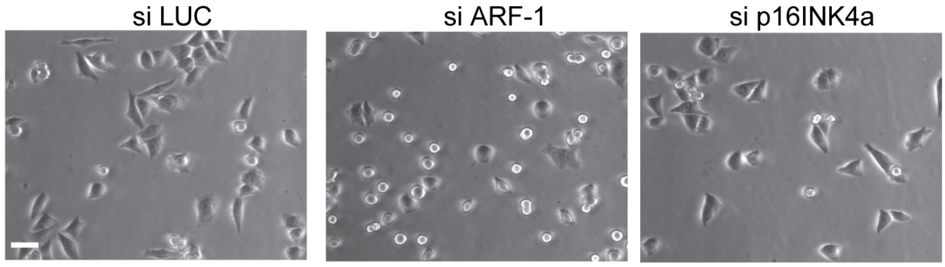

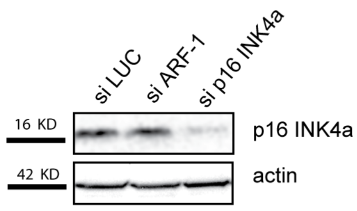

Supplement: Supplementary Figure S4 [file onc2017104x4.pdf]
